# SHR and SCR coordinate root patterning and growth early in the cell cycle

**DOI:** 10.1038/s41586-023-06971-z

**Published:** 2024-01-31

**Authors:** Cara M. Winter, Pablo Szekely, Vladimir Popov, Heather Belcher, Raina Carter, Matthew Jones, Scott E. Fraser, Thai V. Truong, Philip N. Benfey

**Affiliations:** 1 Department of Biology, Duke University, Durham, NC USA; 2 Howard Hughes Medical Institute, Duke University, Durham, NC USA; 3 Translational Imaging Center, Bridge Institute, University of Southern California, Los Angeles, CA USA

**Keywords:** Cell proliferation, Pattern formation, Cell fate, Stem-cell niche, Computational models

## Abstract

Precise control of cell division is essential for proper patterning and growth during the development of multicellular organisms. Coordination of formative divisions that generate new tissue patterns with proliferative divisions that promote growth is poorly understood. SHORTROOT (SHR) and SCARECROW (SCR) are transcription factors that are required for formative divisions in the stem cell niche of *Arabidopsis* roots^1,2^. Here we show that levels of SHR and SCR early in the cell cycle determine the orientation of the division plane, resulting in either formative or proliferative cell division. We used 4D quantitative, long-term and frequent (every 15 min for up to 48 h) light sheet and confocal microscopy to probe the dynamics of SHR and SCR in tandem within single cells of living roots. Directly controlling their dynamics with an *SHR* induction system enabled us to challenge an existing bistable model^3^ of the SHR–SCR gene-regulatory network and to identify key features that are essential for rescue of formative divisions in *shr* mutants. SHR and SCR kinetics do not align with the expected behaviour of a bistable system, and only low transient levels, present early in the cell cycle, are required for formative divisions. These results reveal an uncharacterized mechanism by which developmental regulators directly coordinate patterning and growth.

## Abstract (web summary)

Quantitative time-resolved microscopy analysis of SHR and SCR dynamics in single cells of living *Arabidopsis* roots shows that these transcription factors coordinate formative and proliferative cell divisions early in the cell cycle.

## Main

The final size, shape and function of tissues in multicellular organisms hinge upon the precise control of cell division^4^. Owing to intrinsic and extrinsic cell polarity, a 90° rotation of the division plane determines whether a cell will divide formatively (producing daughter cells with different fates) or proliferatively^5,6^ (producing daughter cells with similar fates). A wrong choice can lead to over-proliferation of cells, resulting in aberrant morphogenesis or tumorigenesis^7,8^. Developmental regulators that specify cell fate and interface directly with the cell cycle machinery^9–11^ are likely arbiters of this decision. However, we have limited knowledge about how these regulators dynamically control cell division in situ.

**Fig. 1:**
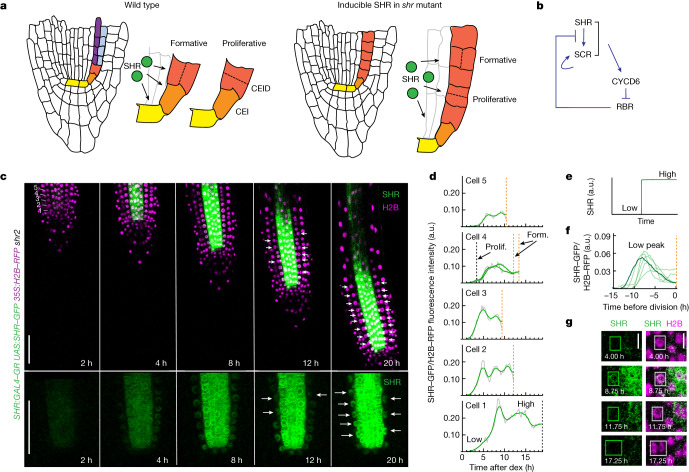
Long-term 4D confocal imaging of SHR reveals dynamics inconsistent with bistability. **a**, Diagram of *Arabidopsis* wild-type and *SHR*:*GAL4–GR* *UAS*:*SHR–GFP* *shr2* mutant roots showing proliferative and formative division planes (adapted from ref. ^52^). SHR moves from the central tissues of the root into the adjacent cell layer. *SHR* expression and formative divisions occur in the inducible line upon treatment with dex. Yellow, QC (quiescent centre); orange, CEI (cortex–endodermal initial); red, CEID (cortex–endodermal initial daughter) and *shr* mutant layer; blue, cortex; purple, endodermis. **b**, Diagram of the SHR–SCR regulatory network controlling formative division based on Cruz–Ramirez et al.^3^. **c**, Confocal median longitudinal sections showing GFP-labelled SHR and H2B–RFP at timepoints after induction with 10 μM dex. Images are representative of independent timecourse experiments with eight roots. Numbers at the top left show the first five cell positions in the mutant ground tissue. Gamma is set to 0.75 to show signal in the mutant layer for the GFP-only images. Top and bottom show different roots. White arrows, formative divisions. Scale bars, 50 μm. **d**, Raw (grey) and smoothed (green) SHR trajectory (SHR–GFP/H2B–RFP fluorescence intensity) over time in the first five cells of a single cell file after full induction (10 μM dex). Plots are representative of 211 cells from independent time courses with 8 roots. Possible low and high steady states are indicated for cell 1. Black dashed line, proliferative division; orange dashed line, formative division. a.u., arbitrary units. **e**, SHR trajectory predicted by the Cruz–Ramirez model showing low and high steady states. **f**, SHR trajectories for cells that show a low peak of SHR accumulation hours prior to dividing formatively. Roots were treated with low dex (0.02 μM or 0.03 μM). Dark green, SHR trajectory corresponding to images in **g**. **g**, Median longitudinal sections through a root tip treated with low dex (0.02 μM) highlighting a cell with a low transient peak of SHR prior to dividing formatively. Plots and images in **f** and **g** are representative of 15 cells from 10 roots showing similar behaviour. Scale bars, 10 μm.

SHR and SCR control the formative division in the *Arabidopsis* root that gives rise to the endodermis and cortex cell types (ground tissue). SHR, a mobile intercellular signalling molecule, is produced in the central tissues of the root and moves outward into adjacent cells, including the endodermis and the cortex–endodermal initial daughter (CEID) cell, where it activates *SCR* expression^12,13^. SHR and SCR together activate the expression of the cell cycle regulator *CYCLIND6;1* (*CYCD6*) only in the CEID, triggering formative division^14^. In *shr* and *scr* mutants, this division does not occur, resulting in a single ground tissue mutant layer, rather than distinct files of endodermis and cortex cells^1,2,15^ (Fig. 1a).

Cruz-Ramírez et al.^3^ proposed a bistable model to explain both how and where SHR and SCR trigger the decision to divide. According to the model, two positive feedback loops generate high stable steady states of SCR and nuclear SHR, triggering formative division (Fig. 1b). Bistability arising from positive feedback is at the heart of mathematical models of decision making in many systems^16^. However, positive feedback does not always lead to bistability^17^, and alternative decision-making mechanisms exist. For example, the simple presence of a factor at the right place and time can alter the cell cycle programme and lead to a different cell fate^11^.

Quantitative time-lapse imaging of transcription factor dynamics has provided key insights into gene-regulatory network function in single cell organisms and mammalian cell lines^18–20^. Assays of multiple transcription factors in tandem on a long timescale can enable examination of their regulatory relationships^21^. However, many technical challenges have made studies of network dynamics in vivo difficult^22^. Phototoxicity and photobleaching, in particular, restrict studies using confocal microscopy to short timescales or infrequent sampling and limit the number of fluorophores that can be imaged simultaneously. Owing to its lower phototoxicity, light sheet microscopy provides the means for longer-term multi-colour imaging of protein dynamics in vivo. This potential has been extolled for nearly two decades, but the technology has been used primarily for observation of cellular dynamics and morphology changes during development^23–25^.

Here, we use long-term 4D imaging of living roots and quantitative analysis to gain insight into the dynamics of the SHR–SCR gene-regulatory network that controls formative divisions in the root stem cell niche. Our measurements revealed a key aspect that was missing from the existing model: namely, that SHR and SCR levels are interpreted within the context of the cell cycle. We present evidence that low threshold levels of SHR and SCR are sufficient and act early in the cell cycle to change the orientation of the division plane.

## SHR dynamics determine formative division

**Extended Data Fig. 1:**
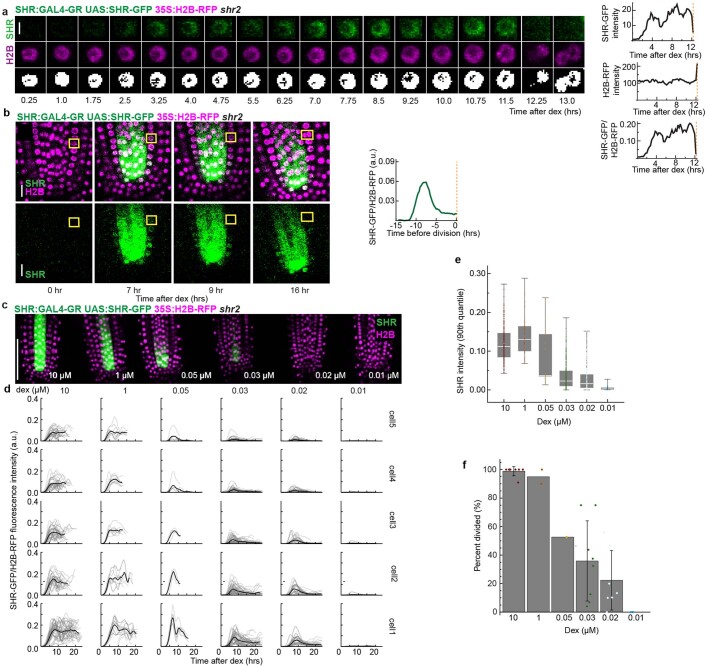
Inducible SHR system produced a variety of protein accumulation trajectories and division outcomes. **a**, Left, raw confocal images of SHR-GFP (green) and H2B-RFP (magenta), and nuclear mask (white; see Supplementary Methods) of a single cell over time. Every third timepoint is shown. Scale bar, 5 μm. Right, quantification of SHR-GFP (top) and H2B-RFP (middle) signal intensities, and the derived SHR trajectory (bottom). SHR-GFP and H2B-RFP signal intensities were extracted from the region demarcated by the nuclear mask at each timepoint. Images and plots are representative of 935 cells from independent timecourse experiments with 29 roots. **b**, Confocal median longitudinal sections through a root tip treated with low dex (0.02 μM) highlighting another cell that divides proliferatively hours after a transient low peak of SHR-GFP is detected. Quantified SHR trajectory is on the right. Scale bar, 10 μm. **c**, Confocal median longitudinal sections acquired 18 hrs after induction with 10, 1, 0.05, 0.03, 0.02, and 0.01 μM dex. Images are representative of 8, 2, 1, 8, 7 and 3 roots for 10, 1, 0.05, 0.03, 0.02 and 0.01 μM dex, respectively. Scale bar, 50 μm. **d**, SHR trajectories for all cells broken out by dex concentration and cell position. SHR trajectories show a quantitative response to different dex concentrations. Grey lines, raw data. Black lines, smoothed averages. 10 μM, *n* = 211 cells from 8 roots; 1 μM, *n* = 63 cells from 2 roots; 0.05 μM, *n* = 25 cells from 1 root; 0.03 μM, *n* = 291 cells from 8 roots; 0.02 μM, *n* = 221 cells from 7 roots; 0.01 μM, 124 cells from 3 roots. **e**, Boxplots of maximum SHR intensity (90th quantile) from all SHR trajectories from all roots treated with different concentrations of dex. Boxes, IQR; centre lines, median, whiskers, full range of the data. **f**, Percentage of cells that divided proliferatively by dex concentration. Data are mean ± s.d. For **e**,**f**, 10 μM, *n* = 158 cells from 8 roots; 1 μM, *n* = 46 cells from 2 roots; 0.05 μM, *n* = 19 cells from 1 root; 0.03 μM, *n* = 236 cells from 8 roots; 0.02 μM *n* = 180 cells from 7 roots; 0.01 μM, *n* = 104 cells from 3 roots.

To investigate the mechanism by which SHR dynamically controls formative cell divisions, we generated a fluorescently labelled inducible SHR construct, *SHR*:*GAL4–GR* *UAS*:*SHR–GFP*, capable of rescuing the formative divisions absent in *shr2* mutants^1,2^ (Fig. 1a). We induced transcription of *SHR–GFP* in its endogenous expression domain (the central tissues of the root; Fig. 1a) using diminishing concentrations of dexamethasone^26^ (dex) (10, 1, 0.05, 0.03, 0.02 and 0.01 μM), and imaged the roots in 3D every 15 min for up to 24 h using confocal microscopy (Fig. 1c and Supplementary Videos 1–4). We observed movement of SHR-GFP into the adjacent mutant ground tissue nuclei, as predicted from previous SHR localization studies^27,28^. We then quantified the fluorescence of SHR–GFP in the ground tissue nuclei (*n* = 935 cells from 29 roots) relative to a nuclei marker as a measure of SHR concentration (Extended Data Fig. 1a and Supplementary Methods), from the time of induction up to formative division or the end of the experiment if no division occurred (Fig. 1d and Supplementary Data 1).

**Extended Data Fig. 2:**
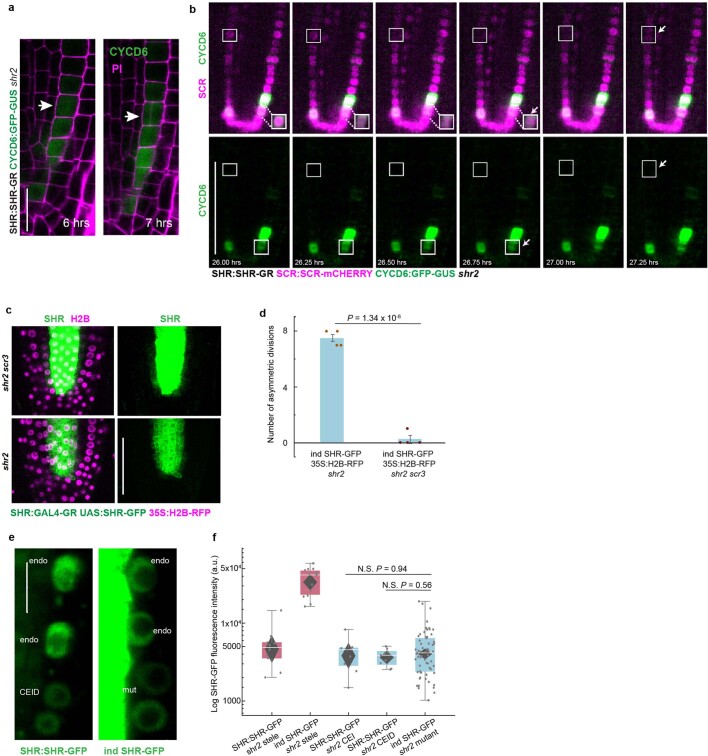
Inducible SHR system controls the SHR-SCR-CYCD6 pathway active in the stem cell niche. **a**, Confocal images of a *SHR:SHR-GR CYCD6:GFP* *shr2* root 6 and 7 h after induction with 10 μM dex. White arrows, cell that divides proliferatively at 7 h. Scale bar, 10 μm. **b**, Confocal images showing *SCR:SCR-mCHERRY* and *CYCD6:GFP-GUS* expression after induction of SHR with 10 μM dex. Maximum pixel intensity for the magenta and green channels is adjusted to enhance visibility of the nucleus in the white box. Inset, image shown with a higher maximum pixel intensity to reduce saturation for that cell. Pink arrow, formatively divided cell; white arrow, proliferatively divided cell. Scale bar, 50 μm. Images in **a**,**b** are representative of independent time courses of 2 roots. **c**, Confocal images of inducible SHR-GFP and H2B-RFP in a *shr2 scr3* (top) or *shr2* (bottom) background after 18-hour 10 μM dex induction. Images are representative of four roots for each mutant line. Scale bar, 50 μm. **d**, Number of formative divisions present in the first five cells of 2 cell files in 6-day old inducible SHR-GFP roots in a *shr2* (*n* = 4 roots) or *shr2 scr3* (*n* = 4 roots) background after 18 h of dex. Unpaired two-sided Student’s t-test *P* is shown. Data are mean ± s.e.m. **e**, Confocal images (green channel only) of a *SHR:SHR-GFP UBQ10:H2B-RFP* *shr2* root (left) and a fully induced (10 μM dex) *SHR:GAL4-GR UAS:SHR-GFP EN7:H2B-RFP* *shr2* (right) root 12 h after dex treatment. Images are representative of 5 and 9 roots of the two respective genotypes. Scale bar, 10 μm. **f**, SHR-GFP fluorescence intensity in the stele (*n* = 10 from 5 roots), CEI (*n* = 9 from 5 roots) and CEID (*n* = 9 from 5 roots) of *SHR:SHR-GFP UBQ10:H2B-RFP* *shr2* plants, and in the stele (*n* = 15 from 8 roots) and mutant cells (*n* = 66 from 9 roots) of *SHR:GAL4-GR UAS:SHR-GFP EN7:H2B-RFP* *shr2* roots 10–15 h after induction with 10 μM dex. Mean SHR-GFP fluorescence in the *shr2* mutant cells prior to formative division in the inducible SHR line is similar to mean levels of SHR-GFP in the CEI and CEID of *shr2* roots. Mann-Whitney two-sided *P* is shown. N.S., not significant. Boxes, IQR; centre lines, mean, whiskers, full range of the data. Source Data

The inducible SHR line enabled us to produce data from many cells in each root, and to produce a variety of SHR accumulation trajectories with different division outcomes (Fig. 1d and Extended Data Fig. 1b–f). We observed near complete rescue of meristematic formative divisions at 1 μM and 10 μM dex (96% and 99%, respectively) and no formative divisions at a concentration of 0.01 μM dex (Extended Data Fig. 1f). Occasionally, a cell divided anticlinally (proliferatively) before the periclinal formative division (Fig. 1d, cell 4). The rescued formative divisions are likely to be controlled by the SHR–SCR–CYCD6 pathway that controls CEID division in the stem cell niche of wild-type plants (Extended Data Fig. 2a–d and Supplementary Note 1), and levels of SHR–GFP in fully induced (10 μM dex) plants were comparable to levels of *SHR:SHR–GFP* in the relevant tissues (Extended Data Fig. 2e,f).

The bistable model^3^ postulates that SHR triggers formative division when nuclear SHR levels in the ground tissue flip to a high steady state (Fig. 1e). Consistent with this model, in many cases we observed a rapid increase in SHR levels followed by a period during which higher SHR levels were relatively constant prior to division (Fig. 1d, cell 2). However, in other cases, a transient low peak of SHR was able to trigger division many hours later (Fig. 1f,g, Extended Data Fig. 1b and Supplementary Video 5). These SHR kinetics are inconsistent with a bistable model in which high steady state levels of nuclear SHR are necessary to trigger division, where the scale of the model (predicted SHR levels) is comparable to the observed range of SHR protein levels (Fig. 1e–g). We sought next to directly examine SHR regulation of SCR levels.

## Bistability is not required for SHR regulation of SCR

**Fig. 2:**
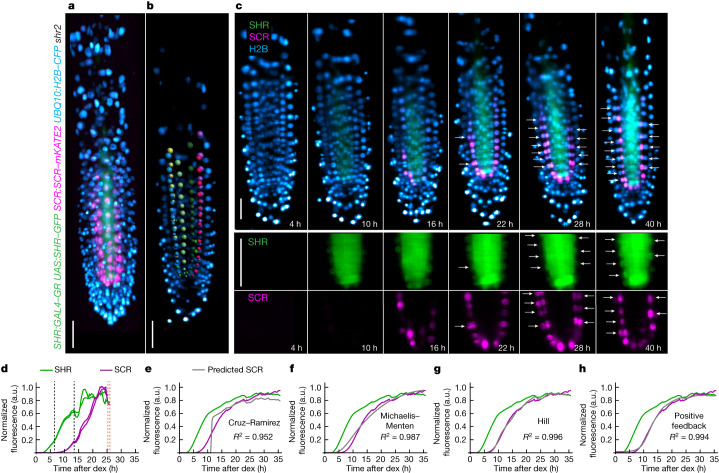
Long-term 4D light sheet imaging of SHR and SCR dynamics reveals that bistability is not required to model their regulatory relationship. **a**, 3D reconstruction of a *z*-stack showing induced SHR–GFP (green), SCR–mKATE2 (magenta) and H2B–CFP (blue) fluorescence in a *SHR*:*GAL4-GR* *UAS*:*SHR–GFP* *SCR*:*SCR–mKATE2* *UBQ10*:*H2B–CFP* *shr2* root. Scale bar, 50 μm. **b**, Endodermal nuclei detected in Imaris are selected for quantification. Colours specify different cell files. Scale bar, 50 μm. **c**, Median longitudinal sections of the root in **a** and **b** showing timepoints after induction with 40 μM dex. Images in **a**–**c** are representative of independent timecourse experiments with nine roots. White arrows, formative divisions. Scale bars, 50 μm. **d**, Quantification of SHR and SCR trajectories (SHR–GFP/H2B–CFP and SCR–mKATE2/H2B–CFP fluorescence intensity, respectively) for a single cell after full induction (40 μM dex). Measurement is representative of 274 cells from independent timecourse experiments with nine roots. SHR and SCR trajectory values are normalized to the 90th quantile (Supplementary Methods). Black dashed line, proliferative division; orange dashed line, formative division. **e**–**h**, Mean of all fully induced SHR (green) and SCR (magenta) normalized trajectories (Supplementary Methods) and predictions for SCR (grey lines) from the Cruz–Ramirez (**e**), Michaelis–Menten (**f**), Hill (**g**) and positive feedback (**h**) models. *R*^2^, adjusted *R* squared; *n* = 274 cells from 9 roots (treated with 40 μM dex; Supplementary Methods).

**Extended Data Fig. 3:**
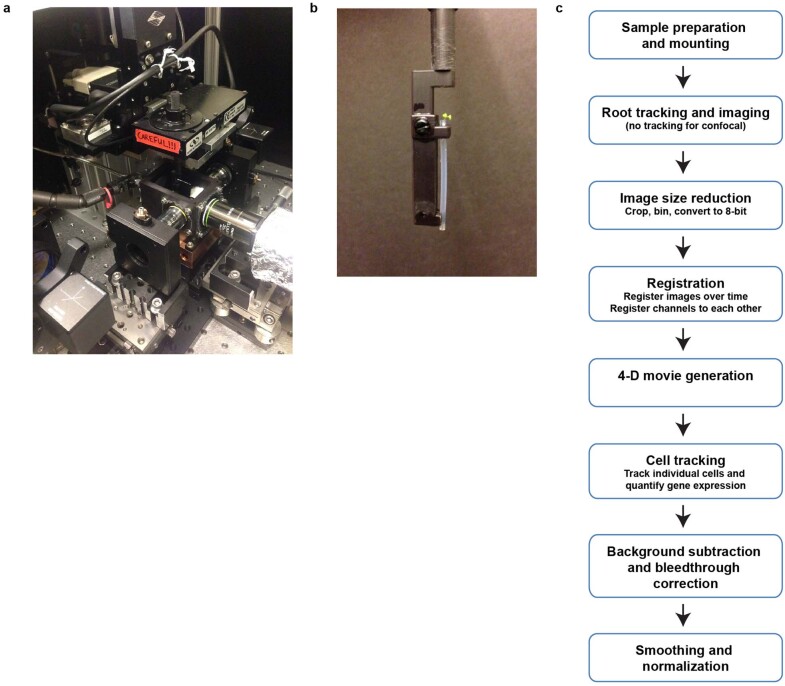
Custom light sheet microscope and analysis pipeline. **a**, Imaging chamber. **b**, Capillary tube containing growing root mounted onto custom holder. The holder is lowered into the imaging chamber for imaging. **c**, Image acquisition and analysis pipeline to produce SHR and SCR trajectories for confocal and light sheet imaging.

We measured SHR and SCR accumulation simultaneously in single nuclei, using a custom light sheet microscope built to image growing root tips under near physiological conditions^29^ (Extended Data Fig. 3a–c, Supplementary Video 6 and Supplementary Methods). We first introduced *SCR*:*SCR–mKATE2* into the *SHR*:*GAL4–GR* *UAS*:*SHR–GFP* *shr2* background. Next, we induced transcription of *SHR–GFP* and subsequent activation of *SCR–mKATE2* expression using different concentrations of dex (40 μM, 20 μM and 0.4 μM; Supplementary Methods) to obtain a range of SHR and SCR accumulation profiles. We imaged and quantified the levels of SHR and SCR in the ground tissue nuclei every 15 min for up to 48 h after induction (Fig. 2a–d, Supplementary Videos 7 and 8 and Supplementary Data 2; *n* = 577 cells from 14 roots). In fully induced roots (40 μM dex), 89% of the meristematic cells had divided after 45 h.

**Extended Data Fig. 4:**
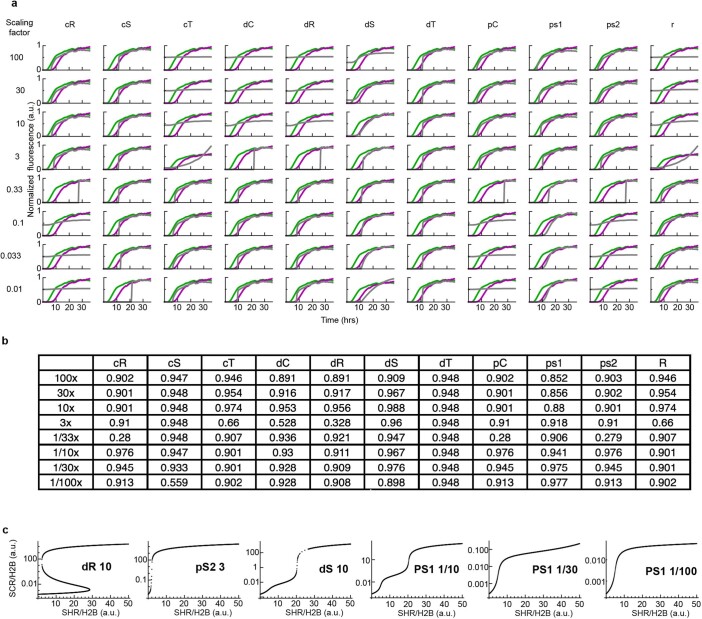
Fitting the SCR data to the Cruz-Ramirez model. **a**, Mean normalized trajectories of SHR-GFP (green) and SCR-mKATE2 (magenta) (*n* = 274 cells from 9 roots treated with 40 μM dex; Supplementary Methods) and predictions of SCR (grey lines) using the Cruz-Ramirez model^3^ (Supplementary Methods) starting from the published Cruz-Ramirez^3^ parameters. Each parameter (columns) was scaled separately by different values (rows). The resulting SCR curve was then scaled to be comparable with the measured SCR curve. **b**, The corresponding adjusted R^2^ for the plots in **a**. **c**, Example steady state plots for SHR/H2B and SCR/H2B using the Cruz-Ramirez model (Supplementary Methods) for six of the parameter sets from **a**. The plot on the left (where the Cruz-Ramirez parameter value for dR was multiplied by 10) shows bistability, while other parameter sets to the right are monostable.

SHR and SCR levels appeared to follow simple dynamics (Fig. 2d). The average SHR and SCR curves closely follow a sigmoid pattern, with SCR having a slightly steeper and later rise (Fig. 2e–h). To further investigate the regulation of SCR by SHR, we fit the data to the bistable model^3^ and to three basic ODE models (Supplementary Methods). For each model, we used the averaged SHR dynamics as input to predict SCR expression. Using the published parameters^3^, the bistable model predicted a rapid jump in SCR levels that we did not observe in the data (Fig. 2e). By varying each parameter by two orders of magnitude, we were able to find parameter regimes that fit the data reasonably well (Extended Data Fig. 4a,b). However, in many of these cases the model no longer displays bistable properties (Extended Data Fig. 4c).

For the three basic ODE models, the fits were comparable (Fig. 2e–h; Michaelis–Menten: adjusted *R*^2^ = 0.987; Hill: adjusted *R*^2^ = 0.996; positive feedback: adjusted *R*^2^ = 0.994). The best fit Hill coefficient for the Hill model was larger than 1, which can occur due to the existence of positive feedback^30^ (Supplementary Methods). This model and the model explicitly incorporating positive feedback visually appeared to capture the rise of SCR better than the Michaelis–Menten model. This finding is consistent with previously described SCR autoregulation^31^.

It is not surprising that we were able to fit the data to the Cruz–Ramirez^3^ model given the larger number of parameters (which can lead to overfitting). However, three other more simple monostable alternatives with fewer parameters also fit the data well. We conclude that bistability is not required to explain the regulatory relationship between SHR and SCR.

## SHR and SCR act early in the cell cycle

**Extended Data Fig. 5:**
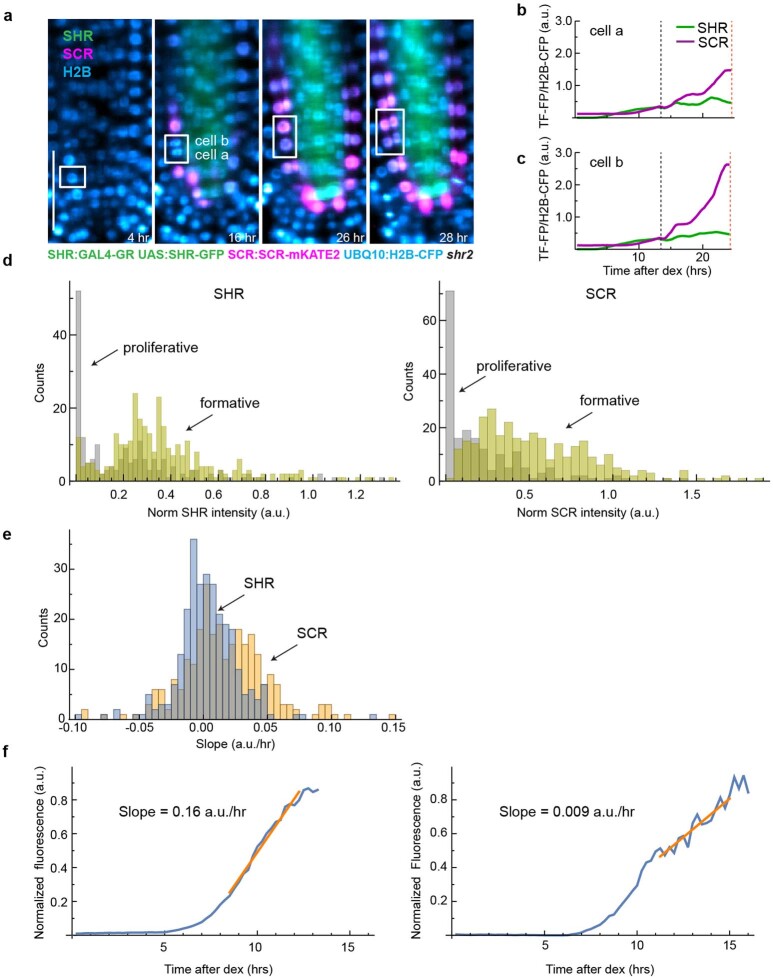
SHR and SCR levels at the time of division are not a critical factor in the decision to divide formatively. **a**, Median light sheet longitudinal sections of a *SHR:GAL4-GR UAS:SHR-GFP SCR:SCR-mKATE2 UBQ10:H2B-CFP* *shr2* root treated with 40 μM dex showing two cells from a common progenitor with different levels of SCR just prior to formative division. Images are representative of 16 cell pairs from independent time courses of 10 roots. Scale bar, 50 μm. **b**,**c**, Quantification of transcription factor fluorescent protein (TF-FP) trajectories for the cells in **a**. **d**, Histograms of the average normalized (Supplementary Methods) SHR and SCR levels found during the last five timepoints of all light sheet SHR and SCR full trajectories (including all dex concentrations). Yellow, formatively dividing cells; grey, proliferatively dividing cells. *n* = 500 cells from independent time courses with 14 roots. **e**, Histograms of the slopes for SHR (blue) and SCR (orange) fully induced (40 μM) trajectories. *n* = 274 cells from independent time courses with 9 roots. **f**, Examples of SCR trajectories (blue) and their fitted slopes (orange). For **e** and **f**, slopes were calculated for trajectories between 1 and 5 h prior to division.

To further investigate the assumption that bistable steady-state levels of SHR and SCR determine whether a cell will divide formatively, we examined SHR and SCR accumulation just prior to division, when the trajectories of both factors have reached high levels. We found variability in SCR levels and did not observe a bimodal distribution corresponding with the fate of the cell (Extended Data Fig. 5a–d). Furthermore, SCR often did not appear to reach a stable point (Extended Data Fig. 5e,f).

**Fig. 3:**
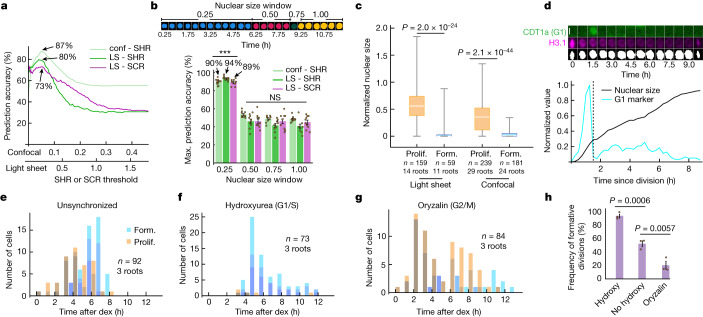
Low threshold levels of SHR and SCR present during an early cell cycle window specify formative division. **a**, Prediction accuracy of trajectory classification into formatively dividing and non-dividing cells for a range of SHR and SCR thresholds (Supplementary Methods). Light sheet (LS), *n* = 449 cells from 14 roots; confocal (conf), *n* = 743 cells from 29 roots. **b**, Maximum prediction accuracy of trajectory classification into proliferatively and formatively dividing cells for a given nuclear size window. ***P = 3.8 × 10^−91^, 8.9 × 10^−61^, 8.0 × 10^−43^ for conf - SHR, LS - SHR and LS - SCR, respectively; one-tailed binomial test. An approximate 50% accuracy is expected by chance. Light sheet, *n* = 500 cells from 14 roots; confocal, *n* = 633 cells from 29 roots. Top, example masks used to calculate the nuclear size trajectory for a single cell. NS, not significant. Data are mean ± s.e.m. **c**, Normalized nuclear size at the beginning of the time course for proliferatively (prolif.) and formatively (form.) dividing cells. Two-tailed Mann–Whitney test. Boxes encompass the IQR, centre lines show the median, and whiskers extend to the full range of the data. **d**, Quantified normalized CDT1a–CFP (G1 marker) fluorescence intensity and normalized nuclear size of a complete cell cycle from a light sheet PlaCCI time course. Top, CDT1a–CFP and H3.1–mCHERRY confocal images showing raw signal, and the Otsu threshold mask. Plot and images are representative of 45 cells from independent timecourse experiments with 2 roots. Scale bar, 5 μm. **e**–**g**, Frequency of dividing cells in unsynchronized roots (**e**), and roots synchronized with hydroxyurea at G1/S (**f**) or oryzalin at G2/M (**g**). Blue, formative; yellow, proliferative. **h**, Percentage of first divisions after dex induction that were formative for roots shown in **e**–**g**. Unpaired one-sided Student’s *t*-test *P* is shown. Data are mean ± s.e.m. Source Data

Therefore, we hypothesized that a threshold amount of SHR and SCR triggers formative division at an earlier timepoint. To test this, we determined the accuracy of predicting formative division across a range of SHR and SCR thresholds (Fig. 3a and Supplementary Methods). Optimal thresholds were low relative to the full range of SHR and SCR levels and were able to accurately predict formative division 80% and 73% of the time, respectively. A similar analysis of the SHR confocal data found a maximum prediction accuracy of 87% (Fig. 3a).

To improve these predictions, we considered the possibility that dynamic features of the SHR and SCR trajectories or position in the cell cycle may contribute to the decision to divide formatively. We took a simple machine learning approach (Supplementary Methods) to determine whether we could predict which cells divide using a set of features describing various aspects of the dynamics of the SHR, SCR and nuclear size trajectories (for example, maximum rate, mean SHR and area under the curve; Supplementary Table 1). We used nuclear size as a proxy for position in the cell cycle^11^ (Fig. 3b and Supplementary Methods). Our learning model was able to predict whether a cell divides formatively 89% and 92% of the time for the light sheet and confocal data, respectively.

To determine the most predictive features, we assessed the ability of each individual feature to discriminate between formatively dividing and non-dividing cells (Supplementary Methods). In addition to features associated with SHR levels, features relating to nuclear size were significant predictors of formative division (Supplementary Tables 2 and 3), suggesting that threshold levels of SHR might be required during a specific window of the cell cycle for formative division to occur.

**Extended Data Fig. 6:**
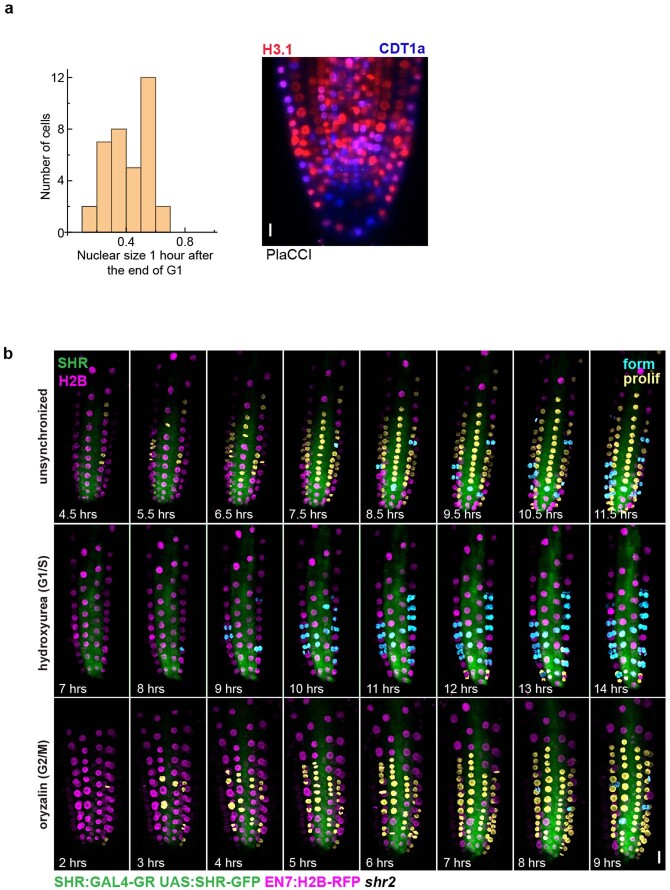
SHR levels are interpreted within the context of the cell cycle. **a**, Left, histogram of normalized nuclear size one hour after the end of G1 (Supplementary Methods; *n* = 50 cells from independent time courses with 2 roots). Right, light sheet image of PlaCCI root showing CDT1a-CFP and H3.1-mCHERRY. Scale bar, 10 μm. **b**, Maximum projection confocal images of unsynchronized (top) and hydroxyurea- (middle) or oryzalin- (bottom) synchronized roots induced with 10 μM dex. Timepoints shown include the 8 h during which most cells first divide. Nuclei of cells are pseudo-colored according to the type of first division after dex treatment. Subsequent divisions maintain the pseudo-colour of the first. Yellow, proliferative division; blue, formative division; green, SHR-GFP; magenta, H2B-RFP expressed from the EN7 promoter. Each row shows a time course of a single root out of 3 roots per condition. Scale bar, 10 μm.

To test this hypothesis, we determined the accuracy of predicting formative versus proliferative division for each individual cell cycle based on whether a threshold of SHR or SCR was reached during one of four quarters of the nuclear size range (Supplementary Methods). Requiring the threshold for SHR or SCR to be met in the first quarter of the nuclear size range resulted in higher accuracies than the predictions using threshold alone (90%, 94% and 89% for the confocal SHR, light sheet SHR and light sheet SCR, respectively) and higher accuracy than requiring the threshold to be met in any of the other three quarters of the nuclear size range (Fig. 3b). In addition, the feature most predictive of formative division over a single cell cycle was the maximum SHR level during the first quarter of the nuclear size range, which accurately predicted 94% and 89% of the confocal and light sheet datasets, respectively (Supplementary Tables 4 and 5). Cells that divided proliferatively had significantly larger nuclei at the beginning of the time course compared with formatively dividing cells (Fig. 3c), suggesting that these cells were already past a critical window of the cell cycle when SHR first reached threshold levels. Using the PlaCCI line^32^, which contains a marker for G1, we found that the 25th percentile of nuclear size falls within G1 or up to 1 h after G1 89% ± 1% of the time (mean ± s.e.m.; *n* = 36 cells from 2 roots) (Fig. 3d, Extended Data Fig. 6a and Supplementary Video 9). This suggests that SHR and SCR are required during G1 or early S phase to trigger a formative division.

**Extended Data Fig. 7:**
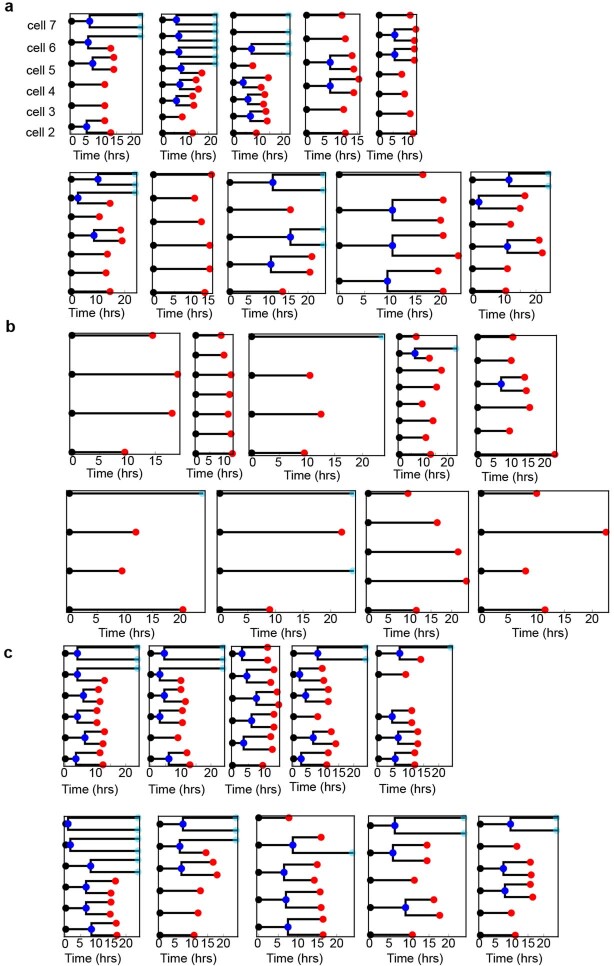
Division trajectories of synchronized cells. Graphical representation showing the timing of proliferative (blue dots) and formative (red dots) divisions for each cell for roots pretreated for 17 h by transfer to plates containing no treatment (**a**), hydroxyurea (**b**), or oryzalin (**c**), followed by transfer to dex for imaging. Each row corresponds to a single root. Data are shown for two roots for each treatment. Each box contains dot plots for cells 2 (bottom) up to 9 (top) from a single cell file. Cells that have not divided proliferatively by the end of the time course have a cyan dot at the last timepoint.

To experimentally validate the hypothesis that position in the cell cycle determines sensitivity to SHR, we synchronized the cell cycle throughout the root meristem prior to induction with dex by treating roots containing the inducible SHR construct with hydroxyurea (see Supplementary Methods), which causes cell cycle arrest at the G1/S transition and early S phase^33^. We anticipated that this treatment would result in larger numbers of cells exposed to SHR during the critical early cell cycle window, leading to greater numbers of formatively dividing cells. Consistent with our hypothesis, 94% ± 3% of the first divisions after dex induction (*n* = 3 roots) in hydroxyurea-treated roots were formative compared to only 52% ± 4% of cells (*n* = 3 roots) treated with dex alone. Conversely, after synchronization with oryzalin at a later stage of the cell cycle (G2/M) followed by dex treatment, only 20% ± 6% of first divisions after dex treatment were formative (Fig. 3e–h, Extended Data Figs. 6b and 7a–c and Supplementary Video 10).

**Extended Data Fig. 8:**
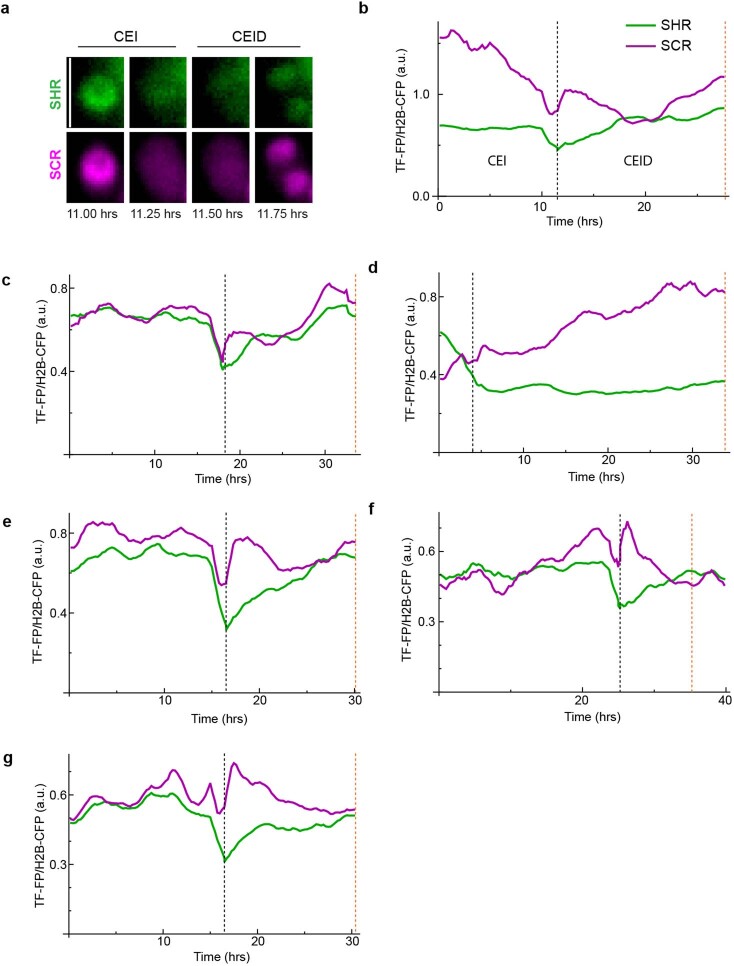
SHR and SCR dynamics in CEI and CEID cells. **a**, Light sheet images of a CEI cell that divides into a CEI and CEID from a time course of a *SHR:SHR-GFP SCR:SCR-mKATE2 UBQ10:H2B-CFP* *shr2* root showing SHR-GFP and SCR-mKATE2 fluorescence just before and after CEI division. Images show a single cell representative of six cells from independent timecourse experiments with three roots. Scale bar, 10 μm. **b**, Normalized SHR-GFP and SCR-GFP accumulation in the CEID shown in **a** and its parent CEI during the time course. **c**–**g**, Additional examples of CEID and parent CEI SHR and SCR trajectories. Orange dashed line, formative division; black dashed line, proliferative division. Cells are from three roots.

To understand how these findings inform division of the CEID in wild-type plants, we investigated the dynamics of SHR–GFP and SCR–mKATE2 driven by their native promoters in plants with a wild-type phenotype. We found that levels of SHR in the CEID fluctuated but never appeared entirely absent. SHR and SCR returned to pre-division levels quickly after division of the CEI (*n* = 6; Extended Data Fig. 8a–g, Supplementary Video 11 and Supplementary Data 3). Thus, it is likely that SHR and SCR are always present early in the cell cycle of the CEID, providing the conditions necessary for formative division.

## Discussion

How developmental regulators control cell division is a central question in developmental biology, with potentially broad applications in understanding basic cell cycle control. Although we cannot exclude the possibility of bistability without a definitive test for hysteresis^34,35^, which would be nearly impossible to perform in our system, our data suggest that SHR and SCR are unlikely to regulate formative cell division through a bistable mechanism. Bistability was proposed to explain how and where the decision to divide formatively is made^3^. Our data suggest an alternative mechanism must exist to restrict SHR–SCR-regulated formative divisions to the CEID in wild-type plants. Levels of SHR and/or other coregulators are possible candidates^36,37^.

Our finding that low transient levels of SHR and SCR can alter the orientation of the division plane is consistent with previous reports^12,31^. A window of sensitivity to these transcription factors in G1 and early S is consistent with the known role of D-type cyclins, including CYCD6, in phosphorylating RBR during the G1/S transition^3^. RBR-associated kinase activity peaks during the G1/S transition and early S phase^38^. Previous studies have suggested that developmental cues specifying division plane orientation are perceived during G1, much earlier than the first visible signs of division plane formation in G2^5^. SHR and SCR are transcription factors that need time to activate their targets in the regulation of division plane orientation. Thus, it may seem obvious they would need to act early in the cell cycle. However, transcription and translation occur on the order of minutes^39^, whereas the median cell cycle time in roots^40^ is approximately 12 h. Given these timescales, SHR and SCR could still function effectively much closer to the time of division plane formation. Understanding the early cellular events regulated by SHR and SCR that lead to the altered division plane is an intriguing avenue for future study.

**Fig. 4:**
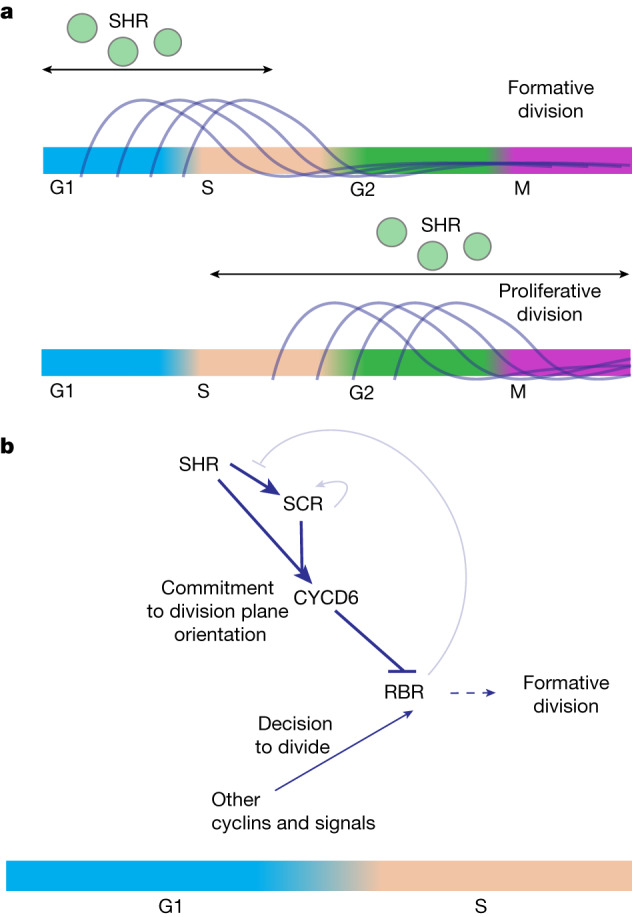
A model for SHR and SCR control of formative division. **a**, Threshold levels of SHR and SCR specify formative division only when present during G1 or early S. **b**, The presence of SHR and SCR during G1 and early S activates CYCD6 to specify the orientation of the division plane, whereas other cyclins and developmental cues commit the cell to division. CYCD6 and other cyclins along with their associated kinases phosphorylate RBR, committing the cell to formative division. The two positive feedback loops (SCR autoregulatory loop and RBR release of SCR after phosphorylation by CYCD6) have a smaller role in the decision to divide formatively than previously predicted^3^.

D-type cyclins activate the RB–E2F bistable switch that commits the cell to DNA synthesis and irreversible progression through mitosis^34^. Notably, however, *SHR* induction cannot initiate formative division outside of the meristem, indicating that SHR is not sufficient to trigger cell division by itself. In addition, CYCD6 is not expressed prior to proliferative divisions in the meristem in our inducible system or in wild-type roots^14^ (Extended Data Fig. 2b). These findings suggest a non-canonical role for SHR, SCR and CYCD6 in determining the orientation of the division plane but not initiation and commitment to division. Thus, the presence or absence of SHR early in the cell cycle determines whether the cell will divide proliferatively or formatively, but other cyclins and other developmental cues must be present to initiate cell cycle progression (Fig. 4a,b). The RB–E2F bistable switch acts to integrate the many signals indicating a cell’s readiness to divide^41^. These findings suggest that both the timing and orientation of cell division are determined there.

The CYCLIN D–RBR–E2F pathway is highly conserved between plants and animals, including humans^42,43^. Coordination of axis determination and cell cycle progression by G1/S regulators is important for formative division in metazoans^44–46^, and D-type cyclins have been implicated in axis determination in metazoans such as *Caenorhabditis elegans*^44^. Thus, our findings may point to a shared mechanism used to coordinate axis and cell fate determination (patterning) with cell cycle progression (growth) across eukaryotic systems. Perturbation of the CYCLIN D–RBR–E2F pathway is estimated to occur during the development of nearly all cancers^47–51^, and defects in division plane orientation and formative division have recently been implicated in the genesis of breast and other cancers^7,8^. Most studies of cell cycle control have used single-cell organisms or cell lines. Future studies using an approach similar to the one described here could reveal mechanisms of cell cycle control that are important during the development of multicellular organisms and suggest opportunities for novel therapeutic interventions in cancer pathogenesis or prevention.

## Methods

All methods are included in the Supplementary Information.

### Reporting summary

Further information on research design is available in the Nature Portfolio Reporting Summary linked to this article.

## Online content

Any methods, additional references, Nature Portfolio reporting summaries, source data, extended data, supplementary information, acknowledgements, peer review information; details of author contributions and competing interests; and statements of data and code availability are available at 10.1038/s41586-023-06971-z.

### Supplementary information

Supplementary Information This file includes Supplementary Note 1, Supplementary Methods, Supplementary Figs 1 and 2 and Supplementary Tables 1–5.

Reporting Summary

Peer Review File

Supplementary Data 1 Raw data extracted from time course images of the *SHR:GAL4-GR UAS:SHR-GFP UBQ10:H2B-RFP* *shr2* line acquired with the confocal microscope (see Methods) and used to create the SHR confocal trajectories.

Supplementary Data 2 Raw data extracted from time course images of the *SHR:GAL4-GR UAS:SHR-GFP SCR:SCR-mKATE2 UBQ10:H2B-CFP* *shr2* line acquired with the light sheet microscope (see Methods), used to create the SHR and SCR light sheet trajectories.

Supplementary Data 3 Raw data extracted from time course images of the *SHR:SHR-GFP SCR:SCR-mKATE2 UBQ10:H2B-CFP* *shr2* line used to create the CEI/CEID trajectories.

Supplementary Video 1 Maximum projection of inducible SHR timecourse. Maximum intensity projection of a confocal time course of a growing *SHR:GAL4-GR UAS:SHR-GFP 35S:H2B-RFP* *shr2* root after induction with 10 μM dex. Magenta, H2B-RFP used for normalization; Green, SHR-GFP.

Supplementary Video 2 Median slices of inducible SHR timecourse. Median longitudinal z-slices of a confocal time course of a growing *SHR:GAL4-GR UAS:SHR-GFP 35S:H2B-RFP* *shr2* root after induction with 10 μM dex. Magenta, H2B-RFP; Green, SHR-GFP.

Supplementary Video 3 Registered median slices of inducible SHR timecourse. Registered median longitudinal z-slices from a confocal time course of a growing *SHR:GAL4-GR UAS:SHR-GFP 35S:H2B-RFP* *shr2* root after induction with 10 μM dex. Magenta, H2B-RFP; Green, SHR-GFP. The white box highlights cell 3 in the left cell file up to an formative division.

Supplementary Video 4 Registered median slices of inducible SHR timecourse – full root. Registered median longitudinal z-slices of a confocal time course of a growing *SHR:GAL4-GR UAS:SHR-GFP 35S:H2B-RFP* *shr2* root after induction with 10 μM dex showing only SHR-GFP along the full length of the root.

Supplementary Video 5 Registered median slices of low dex inducible SHR timecourse. Registered median longitudinal z-slice of a confocal time course of a growing *SHR:GAL4-GR UAS:SHR-GFP 35S:H2B-RFP* *shr2* root after low dex (0.02uM) induction. The SHR levels peak at a low level, go back down, and after several hours the cell divides proliferatively. Corresponds to images shown in Figure 1e. Green, SHR-GFP; Magenta, H2B-RFP.

Supplementary Video 6 3D reconstruction of SCR expression from light sheet images. Reconstruction of a light sheet z-stack in Imaris showing 3D *SCR:SCR-mKATE2* expression in endodermal cells of the root meristem.

Supplementary Video 7 Registered maximum projection of inducible SHR and SCR timecourse. Registered 3D reconstruction of a light sheet time course of a growing *SHR:GAL4-GR UAS:SHR-GFP SCR:SCR-mKATE2 UBQ10:H2B-CFP* *shr2* root after induction with 10 μM dex. The spheres toward the end of the video show the nuclei detected in Imaris that were used for quantification of SHR, SCR, and H2B fluorescence intensity. Cyan, H2B-CFP used for normalization; Green, SHR-GFP; Magenta, SCR-mKate2.

Supplementary Video 8 Registered median slices of inducible SHR and SCR timecourse. Registered median longitudinal z-slices of a light sheet time course of a growing *SHR:GAL4-GR UAS:SHR-GFP SCR:SCR-mKATE2 UBQ10:H2B-CFP* *shr2* root after induction with 10 μM dex. Cyan, H2B-CFP used for normalization; Green, SHR-GFP; Magenta, SCR-mKate2.

Supplementary Video 9 Registered maximum projection of PlaCCI timecourse. Maximum projection of a light sheet time course of a PlaCCI root used to correlate nuclear size with position in the cell cycle. Blue: CDT1a-CFP (G1 marker); Red: H3.1-mCHERRY; Green: CYCB1;1-GFP.

Supplementary Video 10 Registered median slices of inducible SHR timecourses in cell cycle synchronized roots. Registered median longitudinal z-slices of confocal time courses of growing *SHR:GAL4-GR EN7:H2B-RFP* *shr2* roots after induction with 10 μM dex. Roots were pre-treated for 17 hours with 10 μM hydroxyurea (synchronizes cells at G1/S of the cell cycle), 2 μM oryzalin (synchronizes cells at G2/M of the cell cycle) treatment, or a control treatment (transfer to 1/2 MS only plates). The EN7 promoter is active only in the ground tissue, so H2B-RFP is expressed in the mutant ground tissue layer and in the endodermis and cortex after division. Green, SHR-GFP; Magenta, SCR-mKATE2.

Supplementary Video 11 Registered median slices of SHR and SCR timecourse. Registered median longitudinal z-slices of a light sheet time course of a growing *SHR:SHR-GFP SCR:SCR-mKATE2 UBQ10:H2B-CFP* *shr2* root zoomed in to show a dividing CEI cell followed by formative division of the CEID. SHR and SCR levels return quickly to baseline levels after division of the CEI. Formative division of the CEID cell occurs in the last frame. Cyan: H2B-CFP; Green, SHR-GFP; Magenta, SCR-mKATE2.

### Source data

Source Data Fig. 3

Source Data Extended Data Fig. 2

## Data Availability

The pre-processed image files are available in the Duke Digital Research Data Repository (10.7924/r46q2571m; datamanagement@duke.edu). Owing to their large size, original image files are available upon request (please contact researchdatasteward@duke.edu for the first 6 years from publication. To inquire about the availability of this dataset beyond 6 years, please contact caraw97@gmail.com). Complete trajectory data and all metadata needed to run the code are included in the Supplementary material. Source data for figures that were not generated by the code are provided in Excel files. Source data are provided with this paper.

## Code availability

Custom code was central to the conclusions of the paper. The RootTracker (for microscope hardware control), image processing and quantification pipeline, and trajectory data analysis pipeline code are available at 10.5281/zenodo.10044880.
