# Supplementary material for: SHR and SCR coordinate root patterning and growth early in the cell cycle
Source: Nature. 2024 Jan 31;626(7999):611–6. doi: 10.1038/s41586-023-06971-z (PMC10866714; doi:10.1038/s41586-023-06971-z)
Supplement: Supplementary file 1 — This file includes Supplementary Note 1, Supplementary Methods, Supplementary Figs 1 and 2 and Supplementary Tables 1–5. [file 41586_2023_6971_MOESM1_ESM.pdf]

nature portfolio

https://doi.org/10.1038/s41586-023-06971-z

Supplementary information

SHR and SCR coordinate root patterning and growth early in the cell cycle

In the format provided by the authors and unedited

Supplementary Information

SHR and SCR coordinate root patterning and growth early in the cell cycle

Authors:

Cara M. Winter[1,2§], Pablo Szekely[1,2 §], Vladimir Popov[1], Heather Belcher[1], Raina Carter[1], Matthew Jones[3], Scott E. Fraser[3], Thai V. Truong[3], Philip N. Benfey[1,2*]

Table of Contents

Supplementary Methods **2**

Supplementary Note 1. Rescued divisions are likely to be controlled by the SHR-SCR-CYCD6 pathway that controls formative divisions in the stem cell niche. **19**

Supplementary References **20**

Supplementary Figure 1. Correction for maturation time does not alter key conclusions. **23**

Supplementary Figure 2. Supporting figures for Supplementary Methods. **24**

Supplementary Table 1. Quantitative features used to describe SHR, SCR, and nuclear size trajectory dynamics. **25**

Supplementary Table 2. Predictive features from the light sheet data utilizing full trajectories. **34**

Supplementary Table 3. Predictive features from the confocal data utilizing full trajectories. **36**

Supplementary Table 4. Predictive features from the light sheet data utilizing separated trajectories. **37**

Supplementary Table 5. Predictive features from the confocal data utilizing separated trajectories. **39**

Descriptions of Additional Supplementary Information Files. **41**

Supplementary Methods

All methods used in the manuscript are included below.

Construction of Plant Lines

The *Arabidopsis thaliana* accession Columbia (Col-0) was used in this study. The *SHR:GAL4-GR UAS:SHR-eGFP*, *SCR:SCR-mKATE2*, *EN7:H2B-RFP*, and *UBQ10:H2B-CFP* DNA constructs were generated using the Invitrogen MultiSite Gateway® Three-Fragment Vector Construction Kit. To make the *SHR:GAL4-GR UAS:SHR-eGFP* construct, *GAL4-GR UAS* was PCR-amplified from *pDONOR 221 GAL4::GR::UAS::GFP::UAS*[53] and cloned into the D-TOPO vector (Invitrogen). The *SHR* CDS was PCR-amplified from the *pDONR221 SHR* vector, introducing flanking B2 and BamHIXhoI-B3 sequences and cloned into pGEM-T Easy. *eGFP*[54] was also cloned into pGEM-T Easy amplified with flanking BamH1 and XhoI sites. A BamH1-XhoI double digest followed by ligation produced pGEM-T Easy *SHR-eGFP*. *SHR-eGFP* was inserted into P2R-P3 by linearizing pGEM-T Easy *SHR-eGFP* with BsaI followed by a BP reaction. *D-TOPO GAL4-GR UAS* and *P2R-P3 SHR-eGFP* were combined with *pENTR5'SHR* (2.5 kb)[55] into dpGreenBarT[56] in an LR reaction. To make the *SCR:SCR-mKATE2* construct, *mKate2* was amplified from the *pmKATE2-N* plasmid from Evrogen introducing flanking B2 and BamHIXhoI-B3 sequences and cloned into pGEM-T Easy. The 19S terminator was amplified, introducing flanking BamHI and XhoI sites and cloned into pGEM-T Easy. *mKATE2-19S* pGEM-T Easy was generated by a double BamHI XhoI digest of the *mKATE2* and *19S* pGEM-T Easy vectors. *mKATE2-19S* was then inserted into P2R-P3 by a BP reaction. This plasmid was combined with *pDONORP4-P1R SCR2.0*, and *pDONR221 SCR* CDS in an LR reaction into pGII0125[57]. To make *EN7:H2B-RFP* and *UBQ10:H2B-CFP*, the *H2B* CDS from AT5G22880 was amplified and introduced into D-TOPO. This vector, along with *pGEM-P2-sCFP-P3*[58] or *P2R-P3 RFP* was combined with *UBQ10 P4-P1R*[59] or *EN7 P4-P1R* into dpGreenKanT[60]. *RFP* was amplified from *pcDNA3-mRFP* (Addgene 13032) and cloned into P2R-P3 to make *P2R-P3 RFP*. All constructs were transformed into *Arabidopsis* by the floral dip method[61]. The *SHR:GAL4-GR UAS:SHR-eGFP 35S:H2B-RFP shr2* line was created by crossing the *SHR:GAL4-GR UAS:SHR-eGFP shr2/+* line to *35S:H2B-RFP*[62]. The *SHR:GAL4-GR UAS:SHR-eGFP EN7:H2B-RFP shr2* line was created by transforming the *EN7:H2B-RFP* construct into

the *SHR:GAL4-GR UAS:SHR-eGFP shr2/+* line. To make the inducible triple line, *UBQ10:H2B-CFP* was transformed into the *SHR:GAL4-GR UAS:SHR-eGFP shr2/+* line and the resulting line was crossed to *SCR:SCR-mKATE2*. The non-inducible *SHR:SHR-GFP SCR:SCR-mKATE2 UBQ10:H2B-CFP shr2* line was created by crossing *SHR:SHR-GFP shr2*[28] to a *SCR:SCR-mKATE2 UBQ10:H2B-CFP shr2* F2 plant from the cross that generated the inducible triple line. The *SHR:SHR-GR SCR:SCR-mCHERRY CYCD6::GFP-GUS shr2* line was obtained by crossing *SHR:SHR-GR CYCD6::GUS-GFP shr2* [13,63] to *SCR:SCR-mCHERRY*[64]. The *SHR:SHR-GFP UBQ10:H2B-RFP shr2* line was created by crossing *SHR:SHR-GFP shr2*[28] to *UBQ10:H2B-RFP*. All plant materials are available upon request.

Image Acquisition

Confocal Microscopy

Live imaging of the 2-color *SHR:GAL4-GR UAS:SHR-GFP UBQ10:H2B-RFP shr2* and *SHR:GAL4-GR UAS:SHR-GFP EN7:H2B-RFP shr2* roots was performed using an inverted Zeiss 510 Meta confocal microscope. We used Zen 2009 version 6.0.0.303 software for microscope control and data collection.

Confocal sample preparation and mounting

Plants for imaging were grown for 5 days in square Petri dishes containing 1X Murashige and Skoog (MS) 1% sucrose 1% agar media oriented vertically in a Percival in long day growth conditions (16hr/8hr light/dark regime). Prior to imaging, plants were transferred to cell culture/imaging chambers (Thermofisher, Cat. #155360). Roots were covered with a small block of 1% Phytagel containing various concentrations of dex[26]. For hydroxyurea and oryzalin treatments, roots were transferred to MS plates containing 10 µM hydroxyurea (Sigma) for 17 hours or 2 µM oryzalin (Sigma) for 19hrs prior to transfer to an imaging chamber. The chamber was closed with the provided top to prevent water loss. Immersol was used instead of water as the immersion media to prevent evaporation over the time course.

Confocal imaging

We acquired 16-bit images at 512 x 512 resolution with a z-step of 2 µm, 34 slices, and 1x averaging. GFP and RFP were imaged sequentially using the 488 nm and 543 nm excitation lines at 12% and 18% power (15 uW and 7 uW at the sample), respectively, with a Zeiss C-Apochromat 40X 1.2 W Korr water immersion objective (Part #441757-9970). We acquired 4 tiled images every 15 minutes for each experiment for up to 24 hours. The tiles were aligned vertically, and we began each experiment with the root tip located at the top of the first tile. The root grew across the four tiles over the course of the 24-hour time course.

Light Sheet Microscopy

Light sheet imaging of 3-color *SHR:GAL4-GR UAS:SHR-eGFP SCR:SCR-mKATE2 UBQ10:H2B-CFP shr2*, *SHR:SHR-GFP SCR:SCR-mKATE2 UBQ10:H2B-CFP shr2*, and the PlaCCI[32] roots was carried out using a custom-built light sheet microscope (see Light sheet microscope optical setup and Extended Data Figure 3).

Light sheet microscope optical setup

Continuous-wave 488, 457, and 561 nm visible laser light from an Omicron SOLE 6 Compact Laser Light Engine were combined into a single beam and subsequently split and sent into the left and right sides of the imaging chamber, through two illumination objectives (Nikon Plan Fluorite, 10X, NA = 0.3, water dipping)) to bidirectionally illuminate the sample. Pairs of galvanometers (H6215, Cambridge Technologies) upstream of each of the illumination objectives generated a fast-scanned (500 Hz) sheet of light approximately 2 microns thick at the focal point. Fluorescence emitted from the sample was detected through a water-immersion detection objective (Olympus XLUMPLFLN20XW, 20X, NA = 1.0), filtered through detection filters specific to the appropriate fluorophore (Semrock, GFP: FF03-525/50-25; mKATE2: FF01-624/40-25; CFP: FF01-482/35-25) mounted on a filter wheel (Sutter Instruments Lambda 10-3) and a short-pass 750nm filter, and projected onto a cMOS camera (Andor Zyla 5.5) through a double-achromatic tube lens (focal length = 300 mm; Thorlabs ACT508-300-A-ML), to yield a final magnification of 33X. A piezo translational stage (Physiks Instruments P-622.1CD, with controller E-665) controlled the sample's fine z-motion for z-stacking, and a rotational stage (Newport PR50CC, with controller ESP300) controlled the angular position. Both stages are

mounted onto a 3-dimensional stepper-motor stage stackup (Sutter Instrument MPC-200), which provided coarse positioning. A white LED light source (Thorlabs MCWHF2, driver LEDD1) was used to provide ambient light to the plant sample.

Light sheet sample preparation and mounting

Seeds were sterilized with chlorine gas, imbibed and stratified for 2 days prior to planting in FEP (Fluorinated Ethylene Propylene) tubes with an inner diameter of 1/32" and outer diameter of 1/16" (Cole-Parmer, Cat #06406-60). Tubes were cut to 30 mm in length, autoclaved, and filled with 1X MS salt mixture, 1% sucrose, 1% Phytagel media. A hollow channel was created by inserting a 150-micron diameter steel rod into the tubing containing molten Phytagel along the edge of the inner wall. Seeds were planted, embryonic root side down, at the opening of the channel. FEP tubes were attached to the inside of square petri dishes sealed with micropore tape that were oriented vertically in a Percival set to 21°C at ~5500 lux and programmed for long day conditions. Seeds were grown for 5 days prior to imaging. Immediately prior to imaging, FEP tubes were cut to a length of 15 mm, affixed to a custom-designed, 3D-printed sample holder (Extended Data Figure 3b, 3D design files available upon request) and oriented such that the hollow channel containing the root was located closest to the detection objective. The sample holder was lowered into a water-filled chamber such that the roots were vertically oriented. The tip of the FEP tubing was submerged, but the shoot portions of the plant were maintained above the water. For dex treatments, we used different concentrations of dex, as either a continuous treatment or as pulses. A concentrated dex solution was added directly to the water in the chamber and allowed to diffuse into the bottom of the FEP tubing. For continuous treatments (40 µM or 0.04 µM), the dex solution remained in the chamber for the duration of the time course. For the 20 µM and 40 µM dex pulsed treatments, we replaced the media after 1 minute and 5 seconds, respectively. We used a 40 µM dex working concentration in the imaging chamber to get maximal induction of formative divisions in the root tip. The higher concentration of dex relative to that used for the confocal experiments is likely due to the need for diffusion through the imaging chamber and capillary tube in the light sheet sample mounting setup. The ground tissue is patterned correctly in plants with a wild-type phenotype grown on 40 µM dex (Supplementary Figure 2c).

Light sheet imaging

Roots were illuminated bidirectionally using scanned light sheets, creating an optical section parallel to the length of the root. The fluorophores eGFP, sCFP3a, and mKATE2 were imaged using 488, 457, and 561 nm excitation lasers at 10, 10, and 30 percent power, respectively. Laser power measured at the output of the optical fiber was 3.0 mW, 3.1 mW, and 7.4 mW +/- 10% for the 488, 457, and 561 nm lasers, respectively, and the optical throughput is approximately 5% from the fiber output to the sample. Multi-color z-stacks of 130 slices were captured at 15-minute intervals for up to 48 hours using a 300-millisecond exposure time and a step size of 1 micron. Images were captured in 16 bits with a pixel size of 0.197 µm. Plants were illuminated from the top with white light (~140 µmol/m2/sec), synchronized to the data acquisition to turn off during the camera exposure time.

All microscope hardware components (stages, lasers, camera, filter wheel, and white ambient light) were controlled using the RootTracker, a custom-written Java application utilizing the MicroManager core API[65]. Root tips were tracked by shifting the stepper-motor stage positions for the current imaging round by extrapolation using the difference (in x, y, and z) between the centroids of the root tips in the prior two imaging rounds.

Image Analysis

Background-subtracted, smoothed, and normalized protein accumulation trajectories were generated using Fiji 1.50 and 1.52e, and Imaris 9.5.0.

Image pre-processing

Image pre-processing of confocal and light sheet images was automated using a series of custom Fiji plugins that utilized native ImageJ functionality (Extended Data Figure 3c). The 16-bit images were first cropped to reduce black space. Next, pixels were binned 2x2 (light sheet images only) using the ImageJ plugin ‘Binner’ (https://imagej.nih.gov/ij/developer/api/ij/ij/plugin/Binner.html) with the ‘average’ method and converted to 8 bits by mapping pixel values in the range min:max to the range 0:255 (8-bit) using the setMinandMax Fiji function. For the light sheet images, the minimum value (min) was set to the average camera background of 100. The maximum value (max) was set for each channel to minimize saturation in the cells of

interest. The maximum values used for the red, green and blue channels were 2500, 2000 and 2300, respectively. The min and max for the confocal images were set to 0 and 65536.

Images from the nuclei marker channel were registered over time to each other using the PhaseCorrelation function from the Python ImageLib library, which was embedded into a custom ImageJ plugin. Images from all other channels were shifted according to the computed linear shifts. 4D movies were made using Imaris.

Cell tracking and quantification of protein levels

To obtain the trajectories for SHR and SCR, signal intensities for two channels (confocal data) or all three channels (light sheet data) within the nuclear regions of single cell lineages were obtained through one of two automated methods. For the confocal data, we used a custom segmentation and tracking algorithm written in JAVA as Fiji plugins, utilizing native ImageJ functionality to generate a "track" for each cell consisting of a small, cropped image of the nucleus of only that cell at each timepoint. Each image contained the median z-slice through the nucleus at that timepoint. To do this, we used registered hyperstack images containing all timepoints and z-slices for the nuclei channel as input to the algorithm. A square region of interest (ROI) corresponding to the median section of a user-selected cell at timepoint 1 was added to the ImageJ ROI Manager. Additional ROIs for subsequent timepoints were added to the ROI list automatically by finding the median section of the closest cell at the next timepoint. Segmentation and tracking inaccuracies were corrected manually. Nuclei within a track were segmented using the Otsu method[66]. The segmented nucleus image at each timepoint was then used as a mask to extract the pixel intensities of all three channels (Extended Data Figure 3c)

Signal intensities from the light sheet images were obtaining using Imaris software using the Spot Detection and Tracking algorithms. Spot detection and tracking inaccuracies were corrected manually.

We observed that formative divisions do not occur outside of the meristematic zone. Therefore, we quantified SHR and SCR levels only in cells that remained within the meristematic zone for the duration of the time course. This was usually the first five or six cells up from the QC at the

start of the time course. Only cells from the four or five brightest cell files closest to the detectors (confocal) or camera (light sheet) were quantified. Cells directly adjacent to the quiescent center (QC) in position 1 divided later and at a lower frequency than more shootward cells (Supplementary Figure 2a and b). Since QC cells can signal to adjacent cells to maintain them in a non-dividing, undifferentiated state[67], we removed these cells from subsequent analyses.

Background Subtraction and Bleedthrough Correction

To correct for autofluorescence and bleedthrough for each channel for the light sheet experiments, we used the following model,

$$\tilde{G} = G - A_g - BT_g^b - BT_g^r$$
$$\tilde{B} = B - A_b - BT_b^g - BT_b^r$$
$$\tilde{R} = R - A_r - BT_r^g - BT_r^b$$
$$BT_g^b = (B - A_b)\, BT_{ratio}{}_g^b, BT_b^g = \left(B - A_g\right) BT_{ratio}{}_b^g, BT_g^r = 0, BT_b^r = 0$$
$$BT_r^b = 0, BT_r^g = 0$$

where $\tilde{G}, \tilde{B}\ and\ \tilde{R}$ are the corrected signals for the green, blue, and red (corresponding to GFP, CFP, and mKATE2) channels, and $G, B, and\ R$ are the pixel intensities measured via the Spot Detection algorithm in Imaris for the green, blue, and red (corresponding to GFP, CFP, and mKATE2) channels. A_x is the autofluorescence of the root in the *x* channel (where *x* can be *g*, *b*, *r*), and BT_x^y is the bleedthrough of the *y* channel into the *x* channel. Bleedthrough involving the red channels were found to be negligible, hence we set the corresponding parameters to 0.

We took advantage of the fact that *SHR* was not yet induced at the early timepoints and used the average of the first 3 timepoints in the green channel for each cell as the estimate for A_g. The values for A_r were estimated from the image stack at the first timepoint, as the average of the red channel pixel intensities of multiple randomly selected spots within the root tip (but not including the ground tissue). The value for A_b was obtained from roots that did not contain the H2B-CFP marker, as the average of the blue pixel intensities for multiple randomly selected spots within the root tip. The value for $BT_{ratio}{}_g^b$ was empirically determined to be 0.017 based

on images of roots containing only the UBQ10:H2B-CFP construct and imaged with the same settings we used for all experiments. The value for $BT_{ratio}{}_b^g$ was estimated to be 0.137 based on images of induced *SHR:GAL4-GR UAS:SHR-GFP shr2* plants.

For the confocal data, bleedthrough was found to be negligible. Hence, we used the following model to correct for background autofluorescence:

$$\tilde{G} = G - A_g$$
$$\tilde{R} = R - A_r$$

where A_g was estimated as the average of the first 3 timepoints in the green channel for each cell and A_r was estimated from randomly selected regions of roots that did not contain the H2B-RFP marker.

Smoothing and Normalization

We smoothed the data for each cell using a moving average with a window size of 7. On the edges of the time course we used a "reflection" to compensate for missing points in the mean calculation. For Extended Data Figure 1d, smoothed averages of multiple trajectories became increasingly noisy at later timepoints as fewer trajectories remained undivided. We removed these later timepoints by including only timepoints that retained at least 30% of the total trajectories for a given dex concentration and cell position.

Where normalized SHR and SCR trajectories are used, we first divided the SHR-GFP or SCR-mKATE2 fluorescence intensity by the corresponding H2B-RFP or H2B-CFP intensity in that nucleus. Next, for both SHR and SCR trajectories and nuclear size values, we set the minimum value to 0 and the data point at the 90th quantile to 0.9. We used the 90th quantile because it is more robust to noise than the maximal value.

Correction for Maturation Time:

We estimate the typical timescale for the dynamics of the transcription factors to be of the order of magnitude of around several hours (~8hrs). The typical maturation time to reach 90% of the maximal fluorescence is ~1hr for eGFP and ~2hrs for mKate2 at $32°C$ [68]. We estimated the

maturation time at $21°C$ to be ~4hrs to reach 90% (or ~2hrs as the typical maturation time) for mKate2, and ~2hrs (or ~1hr) for the eGFP. These estimations were done by extrapolating the time to reach 90% of maximal fluorescence using the two temperatures that were previously published[68] using an Arrhenius-like equation: $t_{90\%} = Ae^{-k\,T}$, where A is the pre-factor, T is the temperature, and k is the typical rate of change of the function with temperature.

In order to correct for the maturation time we used a simple 1st order ODE model of the dynamics[69] :

$$\frac{d\,D(t)}{dt} = P(t,\theta) - \alpha\,D(t) - \frac{1}{\tau}\,D(t)$$

$$\frac{d\,F(t)}{dt} = \frac{1}{\tau}\,D(t) - \alpha\,F(t)$$

Where D(t) is the "dark" protein, P – is the production term of the protein. We assume that the protein degrades linearly with a rate of α and matures with a typical timescale of τ. F is the fluorescent protein. Using the second equation and the estimated τ, we can extract the value for the dark protein present. The total amount of protein is:

$$T(t) = D(t) + F(t) = \tau\left(\frac{d\,F(t)}{dt} + \alpha\,F(t)\right) + F(t)$$

Therefore, we can estimate the amount of total protein from the measured one. Applying this procedure and estimating α tends to lower the SNR in the data. So, to settle that we used a second smoothing round over the data. The result of the correction for maturation time is a shift of the SHR and SCR trajectories to earlier times, further strengthening our conclusions (Supplementary Figure 1). Because of the extensive data processing that this correction requires we chose to not use this correction throughout the paper.

Data Analysis

Background-subtracted, smoothed, and normalized protein accumulation trajectories were processed and analyzed using Python 3.7, Wolfram Mathematica 13.1, R 3.3.3, Fiji 1.50 and 1.52e, and Imaris 9.5.0.

Full trajectories analyses

SHR and SCR 'full trajectories' were created from the background-subtracted and smoothed measurements of protein levels (see Methods above) for each cell at each timepoint. Timepoints span the range from the start of measurement until either the experiment ended without division (max of 24 hours for the confocal data, 48 hours for the light sheet data) or until a formative division occurred. If a symmetric division occurred along the trajectory, then two full trajectories (from start of measurement to end of experiment or formative division) were created, one for each of the daughter cells. The dynamics prior to the symmetric division were duplicated for each daughter cell. The H2B intensity spikes at the time of division which can cause artifacts in the data. Therefore, if a symmetric division occurred, the H2B value for the time that the symmetric division occurred and the previous timepoint were replaced with the average of the previous few timepoints. To remove artifacts, we trimmed up to 4 timepoints from the beginning and ends of the trajectories. Only full trajectories with more than 10 timepoints were retained for analysis.

Separated trajectories analyses

To test the hypothesis that SHR levels are evaluated based on position in the cell cycle, full trajectories that included one or more symmetric divisions were separated into individual trajectories (referred to as 'separated trajectories') corresponding to a single cell cycle. Only cells that divided formatively or proliferatively were retained for analysis. Cells that did not divide were not included. To remove artifacts, we trimmed up to 4 timepoints from the beginning and ends of the trajectories.

Nuclear size trajectories analyses

A nuclear size trajectory was created for each cell from the quantified nuclear sizes (see Methods below) at each timepoint. A trajectory spans the range from the start of measurement until either the experiment ended without division (max of 24 hours for the confocal data, 48 hours for the light sheet data) or until a formative division occurred. If a symmetric division occurred along the trajectory, then two full nuclear size trajectories (from start of measurement to end of experiment or formative division) were created, one for each of the daughter cells. To do this, the nuclear size dynamics prior to the symmetric division were duplicated for each daughter cell.

Each of the 4 nuclear size windows include the timepoints where the normalized nuclear size falls between 0 – 0.25, 0.25 – 0.5, 0.5 – 0.75, or 0.75 – 1.00. To calculate the normalized nuclear size, for trajectories that comprise a complete cell cycle (from proliferative to formative division or proliferative to proliferative division) we set the minimum and the 90th quantile of the nuclear size to 0 and 0.9, respectively. For full trajectories, or for trajectories that comprise only a part of the cell cycle (from the beginning of the time course to the first division), we set the minimum and 90th quantile of the nuclear sizes for the complete family of trajectories (parent and daughter cells) to 0 and 0.9, respectively.

Calculation of nuclear size

To determine the size of the nucleus for each cell at each timepoint for the confocal data we used a custom Python script to calculate the number of pixels within the circle of the Otsu mask for that nucleus and timepoint. Sometimes, the mask was donut-shaped or was an incomplete circle. To correct for this, we applied a convex hull operation. We first used the OpenCV (version 4.5.3.56) library for Python to calculate the connected components in the mask (two pixels were considered to be touching if they are immediate neighbors horizontally, vertically or diagonally). We filtered the components for those with more than 3 pixels and calculated the convex hull of the new object. This convex hull is considered as the new perimeter of the nucleus. This operation effectively filled in any missing pixels, creating a solid circle. We then calculated the number of white pixels within the circle to determine the size of the nucleus. For the light sheet data, we utilized the 'Diameter' statistic in Imaris for the spot associated with each cell and timepoint.

Modeling and curve fitting

To reduce the noise for modeling, we derived a single average trajectory for SHR and for SCR. We first calculated an average SHR and SCR trajectory for each fully induced (40 μM dex) root for dividing cells. We collected the intensities of the proteins in each cell at each time point and retained only the time points with more than ten measurements to calculate the average for that root. We normalized the dynamics for SHR and SCR for each root as described above. We then manually aligned the different roots to the first inflection point. To obtain one average trajectory

for SHR and another for SCR, we took the average of all the roots by collecting all the values within a 0.25 hr range.

We tested the fit of our average measured SCR dynamics to the predicted SCR dynamics from a modified Cruz-Ramirez model and three new ODE models. The three new models have a linear degradation term and differ only in the production term:

(1) The Michaelis-Menten model:

$$SCR'(t) = \frac{k_{prod} SHR(t)}{k_h + SHR(t)} - \alpha\, SCR(t)$$

Where α is the degradation rate, k_{prod} is the maximal production rate, and k_h is the amount of SHR needed to obtain half the amount of SCR. This is the simplest model, predicting a linear relationship between SHR and SCR production when SHR is low, and saturation of SCR production when SHR is high.

(2) The Hill model:

$$SCR'(t) = \frac{k_{prod} SHR(t)^h}{k_h^h + SHR(t)^h} - \alpha\, SCR(t)$$

Where α is the degradation rate, k_{prod} is the maximal production rate, h is the cooperativity factor, and k_h is the amount of SHR needed to obtain half the amount of SCR. For this generalized Hill function, we found a best-fit Hill coefficient larger than 1 (see below). A high Hill coefficient suggests that the system shows ultrasensitivity, which is an amplified response to a given input[30,70]. Ultrasensitivity could exist for several reasons, including positive feedback[30,70]

(3) And the Positive Feedback model:

$$SCR'(t) = k_{basal} + \frac{k_{prod}\, D\left(SHR(t) SCR(t)\right)}{1 + A\, SHR(t) + B SCR(t) + D\, SHR(t) SCR(t)} - \alpha\, SCR(t)$$

Where α is the degradation rate, k_{prod} is the maximal production rate, k_{basal} is the basal rate of SCR production, and A, B, D are pre-factors of SHR, SCR and the SHR-SCR complex,

respectively. This model explicitly incorporates positive feedback of SCR[12,53] into a Michaelis-Menten-like production term.

For models (2) to (4) we used the average measured SHR(t) curve as input and identified the parameters corresponding to the best fit between the predicted SCR trajectories, SCR(t), and the measured average SCR trajectory. We fit the parameters using a gradient descent method applied to differential equations (https://dpananos.github.io/posts/2019-05-21-odes/).

To determine the model fit, we used the following loss function,

$$\sqrt{\langle (SCR - \widetilde{SCR})^2 \rangle}$$

where SCR denotes the measured SCR curve and $\widetilde{SCR}$ is the estimated SCR curve by the model. When fitting the curves for the Hill model, we used 10 initial condition values for the Hill coefficient, allowing us to reduce the risk of hitting a local minimum in the fitting process and instead reach a global minimum.

We obtained the following best fit parameters:

**Model**	**Parameter**	**Value**
1. Michaelis-Menten	α	0.144091
	kProd	0.913671
	kh	4.97543
2. Hill	α	1.49001
	kProd	1.7844
	kh	0.746098
	h	5.55432
3. Positive Feedback	α	1.4583
	kProd	2.47966
	kBasal	0.0282876
	A	0.102854
	B	1.71131×10^{-6}
	D	1.39126

To assess the goodness of fit we calculated an adjusted R-squared:

$$adjR^2 = \frac{\left(1-(1-R^2)\right)(N-1)}{N-\theta-1}$$

Where: R^2 is the standard measurement of goodness of fit, N is the number of data points and θ is the number of parameters in the model.

Modified Cruz-Ramirez bistable model

The original Cruz-Ramirez model (model 1)[3] includes 6 equations that describe the cytosolic SHR, nuclear SHR, SCR, CYCD6, the active RBR complex, and the SCR-RBR complex. We used it to simulate nuclear SHR behavior for Figure 1e. In all other cases, we used our measured nuclear SHR levels as an input and used the model to predict the SCR output. Therefore, the first two equations are unnecessary to describe this interaction and were removed. This resulted in a model of four ODEs and 11 parameters. Because of the large number of parameters of the bistable model, we used the published model parameters and also varied each parameter individually by two orders of magnitude to fit the model to the data.

Discrimination analysis to identify optimal SHR and SCR thresholds and nuclear size window

To determine the optimal SHR and SCR thresholds for each dataset in Figure 3a, we generated a range of thresholds spanning the range of values present in the data, from 0 to 0.5 and 0 to 2.0 for the confocal and light sheet data, respectively. We then stepped through each threshold and determined its ability to accurately predict whether a cell divided formatively or did not divide. A cell was predicted to divide if the smoothed value from any timepoint was equal to or greater than the threshold. This prediction was compared to the actual fate of the cell (formative division or no division) to determine the prediction accuracy for that threshold. For the nuclear size window analysis in Figure 3e, we tested a range of thresholds (0 to 0.5 and 0 to 2.0 for the confocal and light sheet data, respectively) for each nuclear size window and determined the first timepoint where the SHR/SCR level for each cell crosses each threshold. If that timepoint fell within the time range corresponding to the nuclear size window (0-0.25, 0.25-0.5, 0.5-0.75 or 0.75-1) the cell was predicted to divide formatively. This prediction was compared to the actual fate of the cell (proliferative or formative division) to determine the prediction accuracy for that

threshold and nuclear size window. We determined the optimal threshold based on the highest prediction accuracy. We divided the dataset into 10 groups such that each dex concentration was equally represented in each group. We used the optimal threshold for each nuclear size window to test each group for its prediction accuracy and reported these numbers in Figure 3b. We used full SHR and SCR trajectories for Figure 3a, and the separated trajectories for Figure 3b (see Methods above). We used cells from all dex induction levels in these analyses.

Correlation of nuclear size with position in the cell cycle

To correlate nuclear size with position in the cell cycle, we first identified cells that underwent a complete cell cycle during a time course of the PlaCCI line[32]. We next calculated nuclear size using the H3.1-mCHERRY channel and the method described above. We quantified CDT1a-CFP fluorescence by tracking nuclei using the FIJI plugins described above. We extracted the average CDT1a-CFP signal intensity from the region demarcated by the nuclear mask created using the H3.1-mCHERRY signal. We did not use the H3.1-mCHERRY signal to normalize the CDT1a signal because it varied throughout the cell cycle. For Figure 3d, CDT1a-CFP signal and nuclear size were normalized by setting the min and max values to 0 and 1, respectively. We determined the timepoint corresponding to the end of G1 by first calculating the midpoint between the minimum and the maximum of the CDT1a-CFP fluorescence. We then found the first timepoint after the time of maximum CDT1a-CFP fluorescence that the midpoint value was crossed.

Feature analysis

As a preparation step for feature extraction, we aligned the data by their inflection point, defined as a quarter of the way between the 0.1 and 0.9 quantiles. We removed trajectories of non-divided cells that were shorter than 14 and 38 hours long for the confocal and light sheet data, respectively. We then cropped the remaining trajectories to the aforementioned dimensions. If a cell did not divide by the end of the cropped trajectory, it was labeled as non-divided even if it divided formatively afterward.

We split the data randomly into train (60%) and test (40%) sets and used the training set to find the threshold and the test set to calculate the accuracy. These sets were used throughout.
We defined a set of features to describe various aspects of the aligned trajectory dynamics (see Supplementary Tables 1-5) and found the feature value that maximizes prediction accuracy. For each feature, to determine the value that best separates the formatively dividing and non-dividing cells (full trajectories analysis) or the formatively and proliferatively dividing cells (separated trajectories analysis), we first calculated the mean and standard deviation for each one of the two groups. Next, we defined a range of 3000 steps from the smaller of the two means minus twice its standard deviation to the larger mean plus twice its standard deviation. For each step in the range, we determined how well the value separated the data and returned the best prediction accuracy out of all values tested. We performed two-tailed Mann-Whitney tests for each feature as a significance measure for the separation of the two groups, and used the Benjamini-Hochberg[71] method for FDR correction.

Support vector machine analysis

We used the machine learning algorithm, support vector machine (SVM), to predict the accuracy of separating the data using all the feature values combined. The SVM scores were trained on the training set using the "Classify" function with "SupportVectorMachine" as the method in Wolfram Mathematica. For accuracy prediction we used the ClassifierMeasurement function on the test set.

Statistics and Reproducibility

Images of representative experiments shown in the figures were repeated independently with similar results. The number of times the experiments were executed are shown in parentheses: Figure 1c (8 roots); Figure 1g (15 cells from 10 roots); Figure 2a-c (9 roots); Figure 3d (45 cells from 2 roots); Extended Data Figures 1a (n = 935 cells from 29 roots), Extended Data Figure 1b (15 cells from 10 roots); Extended Data Figure 1c (10 µM: 8 roots; 1 µM: 2 roots; 0.05 µM: 1 root; 0.03 µM,: 8 roots; 0.02 µM,: 7 roots; 0.01 µM: 3 roots); Extended Data Figure 2a (2 roots); Extended Data Figure 2b (2 roots); Extended Data Figure 5a (16 cell pairs from 10 roots); Extended Data Figure 6a (2 roots); Extended Data Figure 6b (3 roots each condition (hydroxy,

oryzalin, unsynchronized)); Extended Data Figure 8a (6 cells from 3 roots); Supplementary Figure 2c (no dex: 8 roots; 40uM dex: 10 roots).

Supplementary Note 1. Rescued divisions are likely to be controlled by the SHR-SCR-CYCD6 pathway that controls formative divisions in the stem cell niche.

CYCD6:GFP expression was observed prior to formative division (18 out of 19 cells from 2 roots) (Extended Data Figure 2a, b). In addition, the rescued formative divisions require SCR (Extended Data Figure 2c, d), could be induced in young plants (D5), and arise from the *shr* mutant layer that exhibits few characteristics of endodermal identity[72], indicating they are unlikely to be controlled by the SCR-independent pathway regulating formative divisions of the endodermis later in development[73].

Supplementary References

53. Moreno-Risueno, M. A. *et al.* Transcriptional control of tissue formation throughout root development. *Science* **350**, 426–430 (2015).
54. Tsien, R. Y. The green fluorescent protein. *Annu. Rev. Biochem.* **67**, 509–544 (1998).
55. Gallagher, K. L., Paquette, A. J., Nakajima, K. & Benfey, P. N. Mechanisms regulating SHORT-ROOT intercellular movement. *Curr. Biol. CB* **14**, 1847–51 (2004).
56. Lee, J.-Y. *et al.* Transcriptional and posttranscriptional regulation of transcription factor expression in Arabidopsis roots. *Proc. Natl. Acad. Sci. U. S. A.* **103**, 6055–6060 (2006).
57. Galinha, C. *et al.* PLETHORA proteins as dose-dependent master regulators of Arabidopsis root development. *Nature* **449**, 1053–1057 (2007).
58. Long, Y. *et al.* Optimizing FRET-FLIM Labeling Conditions to Detect Nuclear Protein Interactions at Native Expression Levels in Living Arabidopsis Roots. *Front. Plant Sci.* **9**, (2018).
59. Van Norman, J. M. *et al.* Periodic root branching in Arabidopsis requires synthesis of an uncharacterized carotenoid derivative. *Proc. Natl. Acad. Sci. U. S. A.* **111**, E1300-1309 (2014).
60. Besnard, F. *et al.* Cytokinin signalling inhibitory fields provide robustness to phyllotaxis. *Nature* **505**, 417–421 (2014).
61. Clough, S. J. & Bent, A. F. Floral dip: a simplified method for Agrobacterium -mediated transformation of Arabidopsis thaliana. *Plant J.* **16**, 735–743 (1998).
62. De Rybel, B. *et al.* A Novel Aux/IAA28 Signaling Cascade Activates GATA23-Dependent Specification of Lateral Root Founder Cell Identity. *Curr. Biol.* **20**, 1697–1706 (2010).

63. Sozzani, R. *et al.* Spatiotemporal regulation of cell-cycle genes by SHORTROOT links patterning and growth. *Nature* (2010) doi:10.1038/nature09143.

64. Clark, N. M. *et al.* Tracking transcription factor mobility and interaction in Arabidopsis roots with fluorescence correlation spectroscopy. *eLife* **5**, e14770.

65. Edelstein, A. D. *et al.* Advanced methods of microscope control using μManager software. *J. Biol. Methods* **1**, e10 (2014).

66. Otsu, N. A Threshold Selection Method from Gray-Level Histograms. *IEEE Trans. Syst. Man Cybern.* **9**, 62–66 (1979).

67. van den Berg, C., Willemsen, V., Hage, W., Weisbeek, P. & Scheres, B. Cell fate in the Arabidopsis root meristem determined by directional signalling. *Nature* **378**, 62–65 (1995).

68. Balleza, E., Kim, J. M. & Cluzel, P. Systematic characterization of maturation time of fluorescent proteins in living cells. *Nat. Methods* **15**, 47–51 (2018).

69. Alber, A. B., Paquet, E. R., Biserni, M., Naef, F. & Suter, D. M. Single Live Cell Monitoring of Protein Turnover Reveals Intercellular Variability and Cell-Cycle Dependence of Degradation Rates. *Mol. Cell* **71**, 1079-1091.e9 (2018).

70. Ferrell, J. E. & Ha, S. H. Ultrasensitivity part I: Michaelian responses and zero-order ultrasensitivity. *Trends Biochem. Sci.* **39**, 496–503 (2014).

71. Benjamini, Y. & Hochberg, Y. Controlling the False Discovery Rate: A Practical and Powerful Approach to Multiple Testing. *J. R. Stat. Soc. Ser. B Methodol.* **57**, 289–300 (1995).

72. Shahan, R. *et al.* A single-cell Arabidopsis root atlas reveals developmental trajectories in wild-type and cell identity mutants. *Dev. Cell* **57**, 543-560.e9 (2022).

73. Paquette, A. J. & Benfey, P. N. Maturation of the Ground Tissue of the Root Is Regulated by Gibberellin and *SCARECROW* and Requires *SHORT-ROOT*. *Plant Physiol.* **138**, 636–640 (2005).

Supplementary Figure 1. Correction for maturation time does not alter key conclusions.

a, Uncorrected (green) and maturation-corrected SHR trajectories (blue) for a single cell file from a confocal time course of a *SHR:GAL4-GR UAS:SHR-GFP UBQ10:H2B-RFP shr2* root. **b-g,** Figures of key findings generated using maturation-corrected trajectories corresponding to Figures 1f (b), 2e-h (c), Extended Data Figure 5d (d), Figure 3a (e), Figure 3b (f) and Extended Data Figure 8b (g).

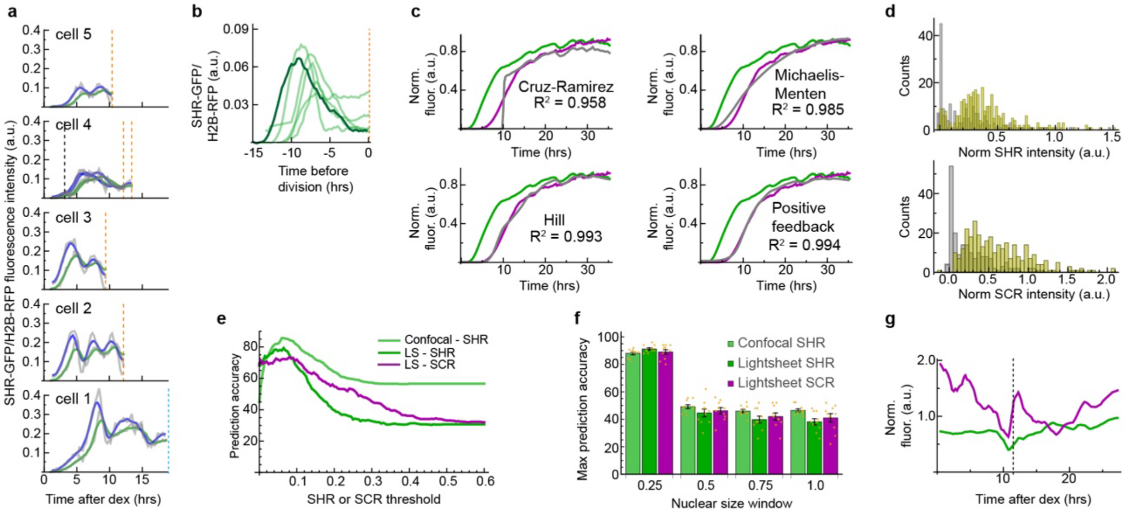

Supplementary Figure 2. Supporting figures for Supplementary Methods.

a, Boxplots of time to division at each cell position for all cells from fully induced (10 µM dex) roots. cell 1, n = 53 cells; cell 2, n = 38 cells; cell 3, n = 41 cells; cell 4, n = 40 cells; cell 5, n = 39 cells. Cells are from 8 roots. Boxes, IQR; centre lines, median, whiskers, full range of the data**. b,** Mean percent of cells divided at each cell position for fully induced roots (10 µM dex). cell 1, n = 53 cells; cell 2, n = 38 cells; cell 3, n = 41 cells; cell 4, n = 40 cells; cell 5, n = 39 cells. Cells are from 8 roots. **c,** Confocal images of roots grown on no dex (left) or 40 µM dex (right) showing correct ground tissue patterning. Images are representative of 8 roots (no dex) and 10 roots (40 µM dex). Scale bar, 50 µm.

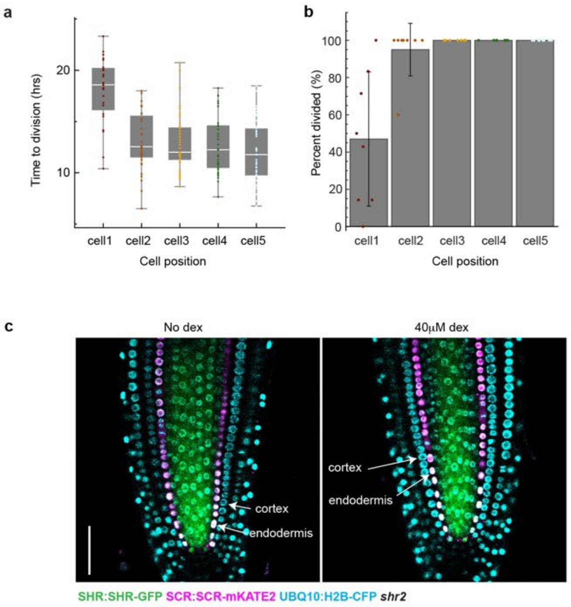

Supplementary Table 1. Quantitative features used to describe SHR, SCR, and nuclear size trajectory dynamics.

Feature Name	Feature Description	Confocal - Full trajectories	Confocal - Separated trajectories	Light sheet - full trajectories	Light sheet - Separated trajectories
time at max rate - shr	time at the maximum rate of the SHR trajectory	X	X	X	X
shr at max rate - shr	SHR level at the time of the maximum rate of the SHR trajectory	X	X	X	X
nuclear size at max rate - shr	size of nucleus at the time of the maximum rate of the SHR trajectory	X	X	X	X
max rate - shr	maximum rate of the SHR trajectory	X	X	X	X
time at max rate - nuclear size	time at the maximum rate of the nuclear size trajectory	X	X	X	X
shr at max rate - nuclear size	SHR level at the time of the maximum rate of the nuclear size trajectory	X	X	X	X
nuclear size at max rate - nuclear size	nuclear size at the maximum rate of the nuclear size trajectory	X	X	X	X
max rate - nuclear size	maximum rate of the nuclear size trajectory	X	X	X	X
time at max rate - norm nuclear size	time at the maximum rate of the normalized nuclear size trajectory	X	X	X	X
shr at max rate - norm nuclear size	SHR level at the time of the maximum rate of the normalized	X	X	X	X

	nuclear size trajectory				
nuclear size at max rate - norm nuclear size	nuclear size at the time of the maximum rate of the normalized nuclear size trajectory	X	X	X	X
max rate - norm nuclear size	maximum rate of the normalized nuclear size trajectory	X	X	X	X
AUC - shr	Area under the curve for the SHR trajectory	X	X	X	X
mean - shr	Mean of the SHR trajectory	X	X	X	X
std - shr	Standard deviation of the SHR trajectory	X	X	X	X
cv - shr	Coefficient of variation of the SHR trajectory	X	X	X	X
mean - nuclear size	Mean of the nuclear size trajectory	X	X	X	X
std - nuclear size	Standard deviation of the nuclear size trajectory	X	X	X	X
cv - nuclear size	Coefficient of variation of the nuclear size trajectory	X	X	X	X
mean - norm nuclear size	Mean of the normalized nuclear size trajectory	X	X	X	X
std - norm nuclear size	Standard deviation of the normalized nuclear size trajectory	X	X	X	X
cv - norm nuclear size	Coefficient of variation of the normalized nuclear size trajectory	X	X	X	X
quantile 0.5 - shr	0.5 quantile of the SHR trajectory	X	X	X	X

first time at quantile 0.5 - shr	first time at the 0.5 quantile of the SHR trajectory	X	X	X	X
quantile 0.95 - shr	0.95 quantile of the SHR trajectory	X	X	X	X
first time at quantile 0.95 - shr	first time at the 0.95 quantile of the SHR trajectory	X	X	X	X
cell pos	cell position relative to the QC (cell 1 is the closest, 5 is the furthest away)	X	X	X	X
quantile 0.3 - nuclear size	0.3 quantile of the nuclear size trajectory	X	X	X	X
first time at quantile 0.3 - nuclear size	first time at the 0.3 quantile of the nuclear size trajectory is crossed	X	X	X	X
quantile 0.5 - nuclear size	0.5 quantile of the nuclear size trajectory	X	X	X	X
first time at quantile 0.5 - nuclear size	first time at the 0.5 quantile of the nuclear size trajectory is crossed	X	X	X	X
quantile 0.7 - nuclear size	0.7 quantile of the nuclear size trajectory	X	X	X	X
first time at quantile 0.7 - nuclear size	first time at the 0.7 quantile of the nuclear size trajectory is crossed	X	X	X	X
quantile 0.95 - nuclear size	0.95 quantile of the nuclear size trajectory	X	X	X	X
first time at quantile 0.95 - nuclear size	first time at the 0.95 quantile of the nuclear size trajectory is crossed	X	X	X	X
quantile 0.3 - norm nuclear size	0.3 quantile of the norm nuclear size trajectory	X	X	X	X
first time at quantile 0.3	first time at the 0.3 quantile of the normalized nuclear	X	X	X	X

- norm nuclear size	size trajectory is crossed				
quantile 0.5 - norm nuclear size	0.5 quantile of the norm nuclear size trajectory	X	X	X	X
first time at quantile 0.5 - norm nuclear size	first time at the 0.5 quantile of the normalized nuclear size trajectory is crossed	X	X	X	X
quantile 0.7 - norm nuclear size	0.7 quantile of the norm nuclear size trajectory	X	X	X	X
first time at quantile 0.7 - norm nuclear size	first time at the 0.7 quantile of the normalized nuclear size trajectory is crossed	X	X	X	X
quantile 0.95 - norm nuclear size	0.95 quantile of the norm nuclear size trajectory	X	X	X	X
first time at quantile 0.95 - norm nuclear size	first time at the 0.95 quantile of the normalized nuclear size trajectory is crossed	X	X	X	X
norm nuclear size at shr threshold	normalized nuclear size at the time the SHR threshold is crossed	X	X	X	X
time from theshold to end	time from threshold to the end of the timecourse	X	X	X	X
norm nuclear size at first shr level of 0.02	normalized nuclear size at the time the SHR trajectory crosses 0.02		X		X
norm nuclear size at first shr level of 0.04	normalized nuclear size at the time the SHR trajectory crosses 0.04		X		
norm nuclear size at first shr level of 0.06	normalized nuclear size at the time the SHR trajectory crosses 0.06		X		

norm nuclear size at first shr level of 0.08	normalized nuclear size at the time the SHR trajectory crosses 0.08		X		
norm nuclear size at first shr level of 0.1	normalized nuclear size at the time the SHR trajectory crosses 0.1		X		X
norm nuclear size at first shr level of 0.2	normalized nuclear size at the time the SHR trajectory crosses 0.2		X		
AUC shr levels at norm nuclear window of 0.25	area under the curve for the SHR trajectory during the normalized nuclear size window 0 - 0.25		X		X
AUC shr levels at norm nuclear window of 0.5	area under the curve for the SHR trajectory during the normalized nuclear size window 0.25 - 0.5		X		X
AUC shr levels at norm nuclear window of 0.75	area under the curve for the SHR trajectory during the normalized nuclear size window 0.5 - 0.75		X		X
AUC shr levels at norm nuclear window of 1	area under the curve for the SHR trajectory during the normalized nuclear size window 0.75 - 1		X		X
Max shr levels at norm nuclear window of 0.25	maximum SHR level during the normalized nuclear size window 0 - 0.25		X		X
Max shr levels at norm nuclear	maximum SHR level during the normalized		X		X

window of 0.5	nuclear size window 0.25 - 0.5				
Max shr levels at norm nuclear window of 0.75	maximum SHR level during the normalized nuclear size window 0.5 - 0.75		X		X
Max shr levels at norm nuclear window of 1	maximum SHR level during the normalized nuclear size window 0.75 - 1		X		X
scr at max rate - shr	SCR level at the time of the maximum rate of the SHR trajectory			X	X
time at max rate - scr	time at the maximum rate of the SCR trajectory			X	X
shr at max rate - scr	SHR level at the time of the maximum rate of the SCR trajectory			X	X
scr at max rate - scr	SCR level at the time of the maximum rate of the SCR trajectory			X	X
nuclear size at max rate - scr	nuclear size at the time of the maximum rate of the SCR trajectory			X	X
max rate - scr	maximum rate of the SCR trajectory			X	X
scr at max rate - nuclear size	SCR level at the time of the maximum rate of the nuclear size trajectory			X	X
scr at max rate - norm nuclear size	SCR level at the time of the maximum rate of the normalized nuclear size trajectory			X	X

AUC - scr	area under the curve for the SCR trajectory			X	X
mean - scr	mean of the SCR trajectory			X	X
std - scr	standard deviation of the SCR trajectory			X	X
cv - scr	coefficient of variation of the SCR trajectory			X	X
quantile 0.5 - scr	0.5 quantile of the SCR trajectory			X	X
first time at quantile 0.5 - scr	first time at the 0.5 quantile of the SCR trajectory			X	X
quantile 0.95 - scr	0.95 quantile of the SCR trajectory			X	X
first time at quantile 0.95 - scr	first time at the 0.95 quantile of the SCR trajectory			X	X
cor shr / scr	correlation between the SHR and SCR trajectories			X	X
norm nuclear size at scr threshold	normalized nuclear size at the SCR threshold			X	X
norm nuclear size at first shr level of 0.05	normalized nuclear size at the first time the SHR level reaches 0.05				X
norm nuclear size at first shr level of 0.075	normalized nuclear size at the first time the SHR level reaches 0.075				X
norm nuclear size at first shr level of 0.3	normalized nuclear size at the first time the SHR level reaches 0.3				X
norm nuclear size at first shr level of 0.5	normalized nuclear size at the first time the SHR level reaches 0.5				X

norm nuclear size at first shr level of 0.7	normalized nuclear size at the first time the SHR level reaches 0.7				X
norm nuclear size at first shr level of 1	normalized nuclear size at the first time the SHR level reaches 1				X
norm nuclear size at first scr level of 0.02	normalized nuclear size at the first time the SCR level reaches 0.02				X
norm nuclear size at first scr level of 0.05	normalized nuclear size at the first time the SCR level reaches 0.05				X
norm nuclear size at first scr level of 0.075	normalized nuclear size at the first time the SCR level reaches 0.075				X
norm nuclear size at first scr level of 0.1	normalized nuclear size at the first time the SCR level reaches 0.1				X
norm nuclear size at first scr level of 0.3	normalized nuclear size at the first time the SCR level reaches 0.3				X
norm nuclear size at first scr level of 0.5	normalized nuclear size at the first time the SCR level reaches 0.5				X
norm nuclear size at first scr level of 0.7	normalized nuclear size at the first time the SCR level reaches 0.7				X
norm nuclear size at first scr level of 1	normalized nuclear size at the first time the SCR level reaches 1				X
AUC scr levels at norm nuclear	area under the curve for the SCR trajectory during the normalized				X

window of 0.25	nuclear size window 0 - 0.25				
AUC scr levels at norm nuclear window of 0.5	area under the curve for the SCR trajectory during the normalized nuclear size window 0.25 - 0.5				X
AUC scr levels at norm nuclear window of 0.75	area under the curve for the SCR trajectory during the normalized nuclear size window 0.5 - 0.75				X
AUC scr levels at norm nuclear window of 1	area under the curve for the SCR trajectory during the normalized nuclear size window 0.75 - 1				X
Max scr levels at norm nuclear window of 0.25	maximum SCR level during the normalized nuclear size window 0 - 0.25				X
Max scr levels at norm nuclear window of 0.5	maximum SCR level during the normalized nuclear size window 0.25 - 0.5				X
Max scr levels at norm nuclear window of 0.75	maximum SCR level during the normalized nuclear size window 0.5 - 0.75				X
Max scr levels at norm nuclear window of 1	maximum SCR level during the normalized nuclear size window 0.75 - 1				X

Supplementary Table 2. Predictive features from the light sheet data utilizing full trajectories.

Feature	Predictive Accuracy*	p-value (Mann-Whitney)**	FDR
std - shr	85.0%	2.07E-11	1.86E-10
quantile 0.95 - shr	84.4%	1.80E-08	8.09E-08
quantile 0.5 - shr	84.4%	9.89E-08	3.90E-07
first time at quantile 0.5 - shr	83.8%	2.61E-04	5.87E-04
mean - shr	83.8%	2.24E-07	7.85E-07
cor shr / scr	82.5%	5.45E-15	5.73E-14
shr at max rate - scr	81.3%	1.21E-09	6.93E-09
max rate - shr	81.3%	7.88E-07	2.48E-06
mean - nuclear size	80.0%	5.09E-18	1.60E-16
norm nuclear size at shr threshold	79.4%	1.01E-09	7.05E-09
quantile 0.7 - nuclear size	79.4%	4.59E-16	5.78E-15
scr at max rate - norm nuclear size	79.4%	7.26E-05	1.83E-04
quantile 0.5 - nuclear size	78.8%	1.95E-16	4.10E-15
quantile 0.3 - nuclear size	78.8%	2.57E-16	4.05E-15
time at max rate - norm nuclear size	78.8%	2.19E-09	1.15E-08
time at max rate - nuclear size	78.8%	2.26E-09	1.10E-08
first time at quantile 0.95 - norm nuclear size	77.5%	4.33E-04	9.10E-04
first time at quantile 0.95 - nuclear size	77.5%	4.33E-04	9.41E-04
nuclear size at max rate - norm nuclear size	77.5%	7.92E-03	1.47E-02
quantile 0.3 - norm nuclear size	76.9%	4.38E-03	8.36E-03
quantile 0.95 - scr	76.9%	9.19E-05	2.15E-04
nuclear size at max rate - scr	76.9%	1.18E-09	7.42E-09
first time at quantile 0.5 - scr	76.3%	2.61E-08	1.10E-07
std - scr	76.3%	1.40E-06	4.02E-06

AUC - scr	76.3%	7.56E-05	1.83E-04
shr at max rate - nuclear size	76.3%	2.67E-05	7.01E-05
max rate - scr	76.3%	1.14E-03	2.24E-03
nuclear size at max rate - shr	76.3%	1.91E-07	7.08E-07
quantile 0.5 - scr	75.6%	2.64E-02	4.38E-02
mean - norm nuclear size	75.0%	9.38E-03	1.64E-02

* Predictive accuracy reflects the ability of a discrimination model to separate the data into formatively divided vs. undivided cells. Only significant (FDR < 0.001) features with a predictive accuracy > 75% are shown.

** Mann-Whitney two-sided test

Supplementary Table 3. Predictive features from the confocal data utilizing full trajectories.

Features	Predictive Accuracy*	p-value (Mann-Whitney)**	FDR
quantile 0.5 - shr	90.8%	6.69E-90	3.01E-88
shr at max rate - nuclear size	87.0%	5.63E-85	8.45E-84
mean - shr	85.4%	2.47E-88	5.56E-87
shr at max rate - shr	85.4%	2.24E-80	2.02E-79
time from theshold to end	85.1%	1.84E-58	9.19E-58
quantile 0.95 - shr	85.1%	2.18E-80	2.46E-79
max rate - shr	84.3%	7.86E-78	5.89E-77
AUC - shr	81.6%	8.31E-75	5.34E-74
norm nuclear size at shr threshold	79.7%	1.99E-52	8.13E-52
cv - shr	79.7%	1.87E-53	8.43E-53
first time at quantile 0.5 - shr	79.3%	7.28E-38	2.52E-37
std - shr	77.4%	7.91E-69	4.45E-68

* Predictive accuracy reflects the ability of a discrimination model to separate the data into formatively divided and undivided cells. Only significant (FDR < 0.001) features with a predictive accuracy > 75% are shown.

** Mann-Whitney two-sided test

Supplementary Table 4. Predictive features from the light sheet data utilizing separated trajectories.

Feature	Predictive Accuracy*	p-value (Mann-Whitney)**	FDR
Max shr levels at norm nuclear window of 0.25	93.5%	1.88E-64	1.79E-62
AUC shr levels at norm nuclear window of 0.25	93.5%	7.44E-63	2.36E-61
Max shr levels at norm nuclear window of 0.5	91.0%	2.44E-64	1.16E-62
AUC shr levels at norm nuclear window of 0.5	91.0%	1.07E-58	2.55E-57
norm nuclear size at shr threshold	90.0%	1.57E-51	1.87E-50
AUC scr levels at norm nuclear window of 0.25	89.5%	9.00E-56	1.42E-54
Max scr levels at norm nuclear window of 0.25	86.5%	1.99E-57	3.78E-56
Max scr levels at norm nuclear window of 0.75	86.0%	1.45E-47	1.37E-46
norm nuclear size at scr threshold	85.5%	6.00E-41	3.80E-40
std - norm nuclear size	84.0%	8.04E-36	3.64E-35
Max shr levels at norm nuclear window of 0.75	82.5%	2.12E-48	2.24E-47
AUC shr levels at norm nuclear window of 0.75	82.0%	5.29E-35	2.18E-34
norm nuclear size at first shr level of 0.02	81.0%	1.01E-28	3.32E-28
cv - norm nuclear size	81.0%	2.78E-45	2.20E-44
quantile 0.95 - scr	80.5%	1.24E-35	5.34E-35
quantile 0.5 - scr	80.5%	6.79E-45	4.61E-44
AUC scr levels at norm nuclear window of 0.75	79.5%	6.27E-35	2.48E-34
AUC - scr	79.5%	1.84E-39	1.09E-38
AUC scr levels at norm nuclear window of 0.5	79.0%	2.13E-47	1.84E-46
norm nuclear size at first shr level of 0.05	79.0%	3.88E-19	9.45E-19
mean - scr	79.0%	5.11E-45	3.74E-44
max rate - norm nuclear size	78.5%	5.81E-38	3.07E-37
Max scr levels at norm nuclear window of 0.5	78.0%	1.42E-52	1.93E-51
scr at max rate - scr	78.0%	1.12E-38	6.26E-38

mean - norm nuclear size	77.5%	1.10E-33	4.17E-33
std - scr	77.0%	2.32E-22	6.48E-22
shr at max rate - norm nuclear size	77.0%	8.83E-10	1.86E-09
scr at max rate - nuclear size	77.0%	9.72E-31	3.55E-30
norm nuclear size at first shr level of 0.075	76.5%	6.38E-15	1.41E-14
nuclear size at max rate - norm nuclear size	76.5%	2.20E-24	6.34E-24
shr at max rate - nuclear size	76.5%	3.30E-29	1.12E-28
scr at max rate - shr	76.5%	1.14E-27	3.48E-27
quantile 0.5 - shr	75.0%	8.34E-38	4.17E-37
AUC - shr	75.0%	6.71E-30	2.36E-29
max rate - scr	75.0%	5.65E-20	1.41E-19

*Predictive accuracy reflects the ability of a discrimination model to separate the data into formatively and proliferatively divided cells. Only significant (FDR < 0.001) features with a predictive accuracy > 75% are shown.

** Mann-Whitney two-sided test

Supplementary Table 5. Predictive features from the confocal data utilizing separated trajectories.

Features	Predictive Accuracy*	p-value (Mann Whitney)**	FDR
Max shr levels at norm nuclear window of 0.25	88.5%	6.92E-85	4.08E-83
AUC shr levels at norm nuclear window of 0.25	86.2%	4.35E-80	8.56E-79
AUC shr levels at norm nuclear window of 0.5	85.4%	3.07E-73	4.53E-72
Max shr levels at norm nuclear window of 0.5	85.0%	2.42E-82	7.13E-81
time from theshold to end	83.0%	1.51E-55	7.42E-55
Max shr levels at norm nuclear window of 0.75	81.8%	1.48E-67	1.09E-66
mean - shr	81.4%	3.97E-70	3.91E-69
AUC - shr	79.8%	4.55E-68	3.83E-67
quantile 0.5 - shr	79.4%	6.40E-72	7.55E-71
shr at max rate - shr	79.4%	1.92E-62	1.26E-61
quantile 0.95 - shr	78.7%	7.70E-60	4.13E-59
shr at max rate - nuclear size	78.7%	3.19E-61	1.88E-60
AUC shr levels at norm nuclear window of 0.75	78.3%	3.65E-53	1.66E-52
cv - norm nuclear size	77.5%	4.31E-53	1.82E-52
std - norm nuclear size	77.5%	1.95E-33	4.99E-33
mean - norm nuclear size	77.1%	5.30E-49	2.09E-48
quantile 0.3 - norm nuclear size	75.9%	1.22E-46	4.50E-46
std - shr	75.9%	2.34E-38	6.92E-38
Max shr levels at norm nuclear window of 0.25	88.5%	6.92E-85	4.08E-83
AUC shr levels at norm nuclear window of 0.25	86.2%	4.35E-80	8.56E-79
AUC shr levels at norm nuclear window of 0.5	85.4%	3.07E-73	4.53E-72
Max shr levels at norm nuclear window of 0.5	85.0%	2.42E-82	7.13E-81
time from theshold to end	83.0%	1.51E-55	7.42E-55
Max shr levels at norm nuclear window of 0.75	81.8%	1.48E-67	1.09E-66

mean - shr	81.4%	3.97E-70	3.91E-69
AUC - shr	79.8%	4.55E-68	3.83E-67
quantile 0.5 - shr	79.4%	6.40E-72	7.55E-71
shr at max rate - shr	79.4%	1.92E-62	1.26E-61
quantile 0.95 - shr	78.7%	7.70E-60	4.13E-59
shr at max rate - nuclear size	78.7%	3.19E-61	1.88E-60
AUC shr levels at norm nuclear window of 0.75	78.3%	3.65E-53	1.66E-52
cv - norm nuclear size	77.5%	4.31E-53	1.82E-52
std - norm nuclear size	77.5%	1.95E-33	4.99E-33
mean - norm nuclear size	77.1%	5.30E-49	2.09E-48
quantile 0.3 - norm nuclear size	75.9%	1.22E-46	4.50E-46
std - shr	75.9%	2.34E-38	6.92E-38

* Predictive accuracy reflects the ability of a discrimination model to separate the data into formatively and proliferatively divided cells. Only significant (FDR < 0.001) features with a predictive accuracy > 75% are shown.

** Mann-Whitney two-sided test

Descriptions of Additional Supplementary Information Files

Supplementary Data 1. Raw data extracted from time course images of the *SHR:GAL4-GR UAS:SHR-GFP UBQ10:H2B-RFP shr2* line acquired with the confocal microscope (see Methods) and used to create the SHR confocal trajectories.

Supplementary Data 2. Raw data extracted from time course images of the *SHR:GAL4-GR UAS:SHR-GFP SCR:SCR-mKATE2 UBQ10:H2B-CFP shr2* line acquired with the light sheet microscope (see Methods), used to create the SHR and SCR light sheet trajectories.

Supplementary Data 3. Raw data extracted from time course images of the *SHR:SHR-GFP SCR:SCR-mKATE2 UBQ10:H2B-CFP shr2* line used to create the CEI/CEID trajectories.

Supplementary Video 1. Maximum projection of inducible SHR timecourse. Maximum intensity projection of a confocal time course of a growing *SHR:GAL4-GR UAS:SHR-GFP 35S:H2B-RFP shr2* root after induction with 10 μM dex. Magenta, H2B-RFP used for normalization; Green, SHR-GFP.

Supplementary Video 2. Median slices of inducible SHR timecourse. Median longitudinal z-slices of a confocal time course of a growing *SHR:GAL4-GR UAS:SHR-GFP 35S:H2B-RFP shr2* root after induction with 10 μM dex. Magenta, H2B-RFP; Green, SHR-GFP.

Supplementary Video 3. Registered median slices of inducible SHR timecourse. Registered median longitudinal z-slices from a confocal time course of a growing *SHR:GAL4-GR UAS:SHR-GFP 35S:H2B-RFP shr2* root after induction with 10 μM dex. Magenta, H2B-RFP; Green, SHR-GFP. The white box highlights cell 3 in the left cell file up to a formative division.

Supplementary Video 4. Registered median slices of inducible SHR timecourse – full root. Registered median longitudinal z-slices of a confocal time course of a growing *SHR:GAL4-GR UAS:SHR-GFP 35S:H2B-RFP shr2* root after induction with 10 μM dex showing only SHR-GFP along the full length of the root.

Supplementary Video 5. Registered median slices of low dex inducible SHR timecourse. Registered median longitudinal z-slice of a confocal time course of a growing *SHR:GAL4-GR UAS:SHR-GFP 35S:H2B-RFP shr2* root after low dex (0.02uM) induction. The SHR levels peak at a low level, go back down, and after several hours the cell divides proliferatively. Corresponds to images shown in Figure 1e. Green, SHR-GFP; Magenta, H2B-RFP.

Supplementary Video 6. 3D reconstruction of SCR expression from light sheet images. Reconstruction of a light sheet z-stack in Imaris showing 3D *SCR:SCR-mKATE2* expression in endodermal cells of the root meristem.

Supplementary Video 7. Registered maximum projection of inducible SHR and SCR timecourse. Registered 3D reconstruction of a light sheet time course of a growing *SHR:GAL4-GR UAS:SHR-GFP SCR:SCR-mKATE2 UBQ10:H2B-CFP shr2* root after induction with 10 µM dex. The spheres toward the end of the video show the nuclei detected in Imaris that were used for quantification of SHR, SCR, and H2B fluorescence intensity. Cyan, H2B-CFP used for normalization; Green, SHR-GFP; Magenta, SCR-mKate2.

Supplementary Video 8. Registered median slices of inducible SHR and SCR timecourse. Registered median longitudinal z-slices of a light sheet time course of a growing *SHR:GAL4-GR UAS:SHR-GFP SCR:SCR-mKATE2 UBQ10:H2B-CFP shr2* root after induction with 10 µM dex. Cyan, H2B-CFP used for normalization; Green, SHR-GFP; Magenta, SCR-mKate2.

Supplementary Video 09. Registered maximum projection of PlaCCI timecourse. Maximum projection of a light sheet time course of a PlaCCI root used to correlate nuclear size with position in the cell cycle. Blue: CDT1a-CFP (G1 marker); Red: H3.1-mCHERRY; Green: CYCB1;1-GFP.

Supplementary Video 10. Registered median slices of inducible SHR timecourses in cell cycle synchronized roots. Registered median longitudinal z-slices of confocal time courses of growing *SHR:GAL4-GR EN7:H2B-RFP shr2* roots after induction with 10 µM dex. Roots were

pre-treated for 17 hours with 10 μM hydroxyurea (synchronizes cells at G1/S of the cell cycle), 2 μM oryzalin (synchronizes cells at G2/M of the cell cycle) treatment, or a control treatment (transfer to 1/2 MS only plates). The *EN7* promoter is active only in the ground tissue, so H2B-RFP is expressed in the mutant ground tissue layer and in the endodermis and cortex after division. Green, SHR-GFP; Magenta, SCR-mKATE2.

Supplementary Video 11. Registered median slices of SHR and SCR timecourse. Registered median longitudinal z-slices of a light sheet time course of a growing *SHR:SHR-GFP SCR:SCR-mKATE2 UBQ10:H2B-CFP shr2* root zoomed in to show a dividing CEI cell followed by formative division of the CEID. SHR and SCR levels return quickly to baseline levels after division of the CEI. Formative division of the CEID cell occurs in the last frame. Cyan: H2B-CFP; Green, SHR-GFP; Magenta, SCR-mKATE2.
