# Supplementary material for: SHR and SCR coordinate root patterning and growth early in the cell cycle
Source: Nature. 2024 Jan 31;626(7999):611–6. doi: 10.1038/s41586-023-06971-z (PMC10866714; doi:10.1038/s41586-023-06971-z)
Supplement: Supplementary file 2 — Reporting Summary [file 41586_2023_6971_MOESM2_ESM.pdf]

nature portfolio

Corresponding author(s): Cara Winter and Pablo Szekely

Last updated by author(s): Dec 12, 2023

Reporting Summary

Nature Portfolio wishes to improve the reproducibility of the work that we publish. This form provides structure for consistency and transparency in reporting. For further information on Nature Portfolio policies, see our Editorial Policies and the Editorial Policy Checklist.

Statistics

For all statistical analyses, confirm that the following items are present in the figure legend, table legend, main text, or Methods section.

n/a	Confirmed	
☐	☒	The exact sample size (n) for each experimental group/condition, given as a discrete number and unit of measurement
☐	☒	A statement on whether measurements were taken from distinct samples or whether the same sample was measured repeatedly
☐	☒	The statistical test(s) used AND whether they are one- or two-sided<br>*Only common tests should be described solely by name; describe more complex techniques in the Methods section.*
☐	☒	A description of all covariates tested
☐	☒	A description of any assumptions or corrections, such as tests of normality and adjustment for multiple comparisons
☐	☒	A full description of the statistical parameters including central tendency (e.g. means) or other basic estimates (e.g. regression coefficient) AND variation (e.g. standard deviation) or associated estimates of uncertainty (e.g. confidence intervals)
☐	☒	For null hypothesis testing, the test statistic (e.g. F, t, r) with confidence intervals, effect sizes, degrees of freedom and P value noted<br>*Give P values as exact values whenever suitable.*
☒	☐	For Bayesian analysis, information on the choice of priors and Markov chain Monte Carlo settings
☒	☐	For hierarchical and complex designs, identification of the appropriate level for tests and full reporting of outcomes
☒	☐	Estimates of effect sizes (e.g. Cohen's d, Pearson's r), indicating how they were calculated

Our web collection on statistics for biologists contains articles on many of the points above.

Software and code

Policy information about availability of computer code

Data collection	For data collection on the confocal microscope, we used Zen 2009 version 6.0.0.303. For data collection on the light sheet microscope we used a custom-written Java application called RootTracker (https://doi.org/10.5281/zenodo.10044880).
Data analysis	Data were processed and analyzed using Imaris 9.5.0 and custom scripts written in Python 3.7, Wolfram Mathematica 13.1, R 3.3.3, Fiji 1.50 and 1.52e). Trajectory analysis using Mathematica is available here: https://doi.org/10.5281/zenodo.10044880.

For manuscripts utilizing custom algorithms or software that are central to the research but not yet described in published literature, software must be made available to editors and reviewers. We strongly encourage code deposition in a community repository (e.g. GitHub). See the Nature Portfolio guidelines for submitting code & software for further information.

Data

Policy information about availability of data

All manuscripts must include a data availability statement. This statement should provide the following information, where applicable:

- Accession codes, unique identifiers, or web links for publicly available datasets
- A description of any restrictions on data availability
- For clinical datasets or third party data, please ensure that the statement adheres to our policy

The pre-processed image files are available in the Duke Digital Research Data Repository (https://doi.org/10.7924/r46q2571m; datamanagement@duke.edu). Due to their large size, original image files are available upon request (Please contact researchdatasteward@duke.edu for the first 6 years from publication. To inquire

about the availability of this dataset beyond 6 years, please contact caraw97@gmail.com.) Complete trajectory data and all metadata needed to run the code are included in the Supplementary material. Source data for figures that were not generated by the code are provided in Excel files.

Human research participants

Policy information about studies involving human research participants and Sex and Gender in Research.

Reporting on sex and gender	NA
Population characteristics	NA
Recruitment	NA
Ethics oversight	NA

Note that full information on the approval of the study protocol must also be provided in the manuscript.

Field-specific reporting

Please select the one below that is the best fit for your research. If you are not sure, read the appropriate sections before making your selection.

☒ Life sciences ☐ Behavioural & social sciences ☐ Ecological, evolutionary & environmental sciences

For a reference copy of the document with all sections, see nature.com/documents/nr-reporting-summary-flat.pdf

Life sciences study design

All studies must disclose on these points even when the disclosure is negative.

Sample size	Sample sizes are based on number of roots or number of cells, indicated in the text. The number of cells quantified within one root was determined by how many cells stayed within the meristem for the duration of the timecourse, and were present on the brightest side of the root which was the side closest to the detector (confocal) or camera (light sheet). We made every attempt to replicate every condition with multiple roots, but due to the challenges associated with long-term timecourse imaging and with maintaining a custom-built microscope, this was not always possible. In the two dex conditions where only one root is represented in the data, the data obtained from those follow the trends that are apparent from the other conditions with replicates.
Data exclusions	Roots that were not imaged with the same imaging conditions were removed from statistical analyses. Roots that were not imaged successfully for the complete timeperiod (22hrs for the confocal, 45hrs for the light sheet) were excluded.
Replication	All conditions were replicated with multiple cells. These came from multiple roots where possible. Due to the challenges of imaging, data from two low dex condition came from single roots. The number of roots and cells used are indicated in the figure legends for each analysis.
Randomization	Roots were imaged on different days. All plants were grown under identical growth conditions. All roots of a given genotype were taken from a single transgenic line (single genomic insertion location).
Blinding	Investigators performing cell tracking and quantification of gene expression were blind to the dex concentration of the treatment.

Reporting for specific materials, systems and methods

We require information from authors about some types of materials, experimental systems and methods used in many studies. Here, indicate whether each material, system or method listed is relevant to your study. If you are not sure if a list item applies to your research, read the appropriate section before selecting a response.

Materials & experimental systems

n/a	Involved in the study	
☒	☐	Antibodies
☒	☐	Eukaryotic cell lines
☒	☐	Palaeontology and archaeology
☒	☐	Animals and other organisms
☒	☐	Clinical data
☒	☐	Dual use research of concern

Methods

n/a	Involved in the study	
☒	☐	ChIP-seq
☒	☐	Flow cytometry
☒	☐	MRI-based neuroimaging
